# A Novel Perspective on Lead-Induced Protamine-like Protein-DNA Interactions in *Mytilus galloprovincialis*: A Molecular and Computational Study

**DOI:** 10.3390/biom16040529

**Published:** 2026-04-02

**Authors:** Carmela Marinaro, Simona Amore, Rosaria Garofalo, Barbara Sebastiano, Giulio Santaniello, Simona Cafaro, Donato Sansone, Carmen Di Giovanni, Gennaro Lettieri, Marina Piscopo

**Affiliations:** 1 Department of Biology, University of Naples Federico II, 80126 Naples, Italy; 2 National Reference Centre for the Analysis and Study of Correlations Between the Environment, Animals and Humans, Experimental Zooprophylactic Institute of Southern Italy, Via Salute 2, 80055 Portici, Italy; 3 Drug Discovery Laboratory, Department of Pharmacy, University of Naples Federico II, 80131 Naples, Italy; 4 Department of Life Sciences, Health and Health Professions, Link Campus University, 00165 Rome, Italy

**Keywords:** lead toxicity, protamine-like, *Mytilus galloprovincialis*, reproductive health, protamine-like-DNA interaction, Pb-protamine-like interactions, lead-DNA interactions, structural modeling, environmental pollutants

## Abstract

*Mytilus galloprovincialis* is a significant indicator species due to its ability to bioaccumulate environmental pollutants, such as lead (Pb), which can hinder essential reproductive molecular processes. This study aimed to examine the effect of exposure to lead (0.5, 1.5 and 5 μg/L PbCl_2_) on the state of protamine-like (PL) proteins—the primary components of sperm nuclear basic proteins—and their interaction with DNA. PL proteins were analysed using acetic acid–urea PAGE and SDS-PAGE, after which their ability to bind and protect DNA from oxidative damage was also assessed. Exposure to lead resulted in SDS-PAGE-detectable alterations of the PL, particularly at levels of 1.5 µg/L and 5 µg/L of PbCl_2_ and modified their capacity for DNA-binding at all doses of PbCl_2_. Experiments testing the release of PLs from sperm nuclei further confirmed this, revealing a reduced release. In addition, the ability of PL proteins to protect DNA from oxidative damage was reduced at the highest exposure dose, suggesting improper condensation of sperm chromatin. Computational analyses of human protamines in the presence of lead indicated the formation of coordination complexes with Pb^2+^ in PLI-II and PL-III, potentially impairing DNA binding. Overall, our study demonstrates that exposure to lead alters the function of PL proteins and potentially destabilises the sperm chromatin of *M. galloprovincialis*. This provides valuable insights into the reproductive toxicity of this metal.

## 1. Introduction

Environmental pollution in the Mediterranean Sea represents a pressing challenge, stemming from both historical practices and ongoing anthropogenic activities. Lead (Pb) contamination constitutes a significant concern: the historical use of leaded gasoline until 2003 in countries such as Italy, Spain, and Greece has significantly elevated levels of this metal in coastal and marine environments [1,2,3,4]. Although recent trends indicate a reduction in surface water Pb concentrations, which have halved in the last decade [5,6], sediments that have accumulated lead over the past 150 years now act as reservoirs, releasing the contaminant into the overlying water column and complicating remediation efforts [7]. Studies reveal that anthropogenic Pb signals persist even at depths of approximately 1000 m [8], highlighting the enduring impact of human activities dating back to ancient civilizations [9]. Pb enters the marine environment through various anthropogenic sources, including industrial processes, mining activities, agricultural runoff, and vehicular emissions [10,11,12,13,14,15]. Background dissolved Pb in open Mediterranean waters falls in the low nanogram-per-litre range following the phase-out of leaded gasoline, which reduced dissolved Pb in Italian coastal seawater by ~50% between 2000 and 2004 [5]. However, these values are not representative of urbanized coastal environments such as the Gulf of Naples, a well-documented heavy-metal hotspot where significant Pb enrichment in waters and sediments results from industrial discharges, maritime traffic, urban runoff, and inputs from the Sarno River [16]. Elevated heavy metal concentrations at multiple stations within the Gulf have been further confirmed by long-term regional monitoring (ARPAC, 2013–2019) [17]. Once in the ecosystem, this contaminant poses significant risks to marine biodiversity, as marine ecosystems serve as sinks for terrestrial contaminants, leading to bioaccumulation in key organisms such as invertebrates [18,19]. Among these, *M. galloprovincialis* is widely used as a bioindicator for marine pollution.

It is well documented [20,21] that exposure to heavy metals negatively influences the reproductive performance of this species, causing significant cellular disorders, maturation arrests, and loss of germ cells during spermatogenesis [22,23,24]. Lead is a “soft” Lewis acid with a larger ionic radius (1.19 Å, ~20% larger than Zn^2+^ or Mg^2+^) and a stereochemically active lone pair, which allows it to bind to electronegative atoms such as sulfur, oxygen, and nitrogen in DNA bases and protein-DNA binding domains. Its high coordination number and chemical softness make it highly thiophilic, favoring interactions with cysteine-rich sites in zinc-finger proteins, while the lone pair often leads to hemidirected, non-symmetrical coordination geometries. These properties allow Pb^2+^ to bind tightly but improperly, destabilizing the normal protein-DNA interaction. Specifically, Pb^2+^ competes with Zn^2+^ in DNA-binding proteins, such as zinc fingers, efficiently displacing Zn from cysteine or histidine ligands. Unlike Zn^2+^, Pb^2+^ does not stabilize the proper protein fold, leading to loss of transcriptional activity [25,26,27,28,29,30,31,32]. Similarly, Pb^2+^ can compete with Mg^2+^ in stabilizing the DNA phosphate backbone, binding with high affinity to phosphates and nucleobase carbonyls, but often distorting the DNA helix, inducing local strand breaks or structural transitions, and forming so-called leadzyme complexes capable of site-specific RNA hydrolysis. In addition, Pb^2+^ can bind via the minor groove of DNA, creating electrostatic perturbations that further destabilize chromatin and inhibit the binding of transcription factors to gene promoters. Overall, the combination of high thiophilicity, larger size, hemidirected coordination, and strong phosphate/nucleobase affinity renders Pb^2+^ uniquely effective at disrupting both protein-DNA and DNA structural interactions compared to other divalent cations [33,34,35,36]. In addition, Pb is capable of mimicking essential metals such as calcium and zinc, interfering with cellular signaling pathways and enzymatic activities crucial for cellular integrity [37] and inducing oxidative stress [38,39,40,41], inflammation, and DNA damage [42]. Moreover, Pb is highly toxic to mussel spermatozoa because it causes DNA damage and increases oxidative stress and severe damage [43,44]. These effects reduce spermatozoa motility and produce damaged membranes [43] through alterations of energy metabolism (cAMP and Ca^2+^). All these effects compromise fertilisation success and negatively impact mussel populations [45,46,47,48,49,50]. Of particular relevance to reproduction is the effect of Pb on sperm chromatin. In *M. galloprovincialis*, sperm chromatin is primarily structured by three “protamine-like” (PL) proteins: PL-II, PL-III, and PL-IV [51]. These proteins play a crucial role in chromatin condensation and the protection of paternal DNA. Recent studies have highlighted that Pb exposure alters the expression and functionality of PL proteins, leading to changes in chromatin structure that can compromise sperm viability and fertilization success [14]. Despite the growing awareness of the environmental implications of Pb, gaps remain in understanding the specific molecular mechanisms through which this metal affects the interaction between PL proteins and DNA, influencing sperm chromatin organization in *M. galloprovincialis*. To this end, this study examined the impact of Pb on the reproductive health of this organism, with a particular focus on possible changes to protamine-like proteins and their ability to bind to and defend DNA from oxidative damage. This was performed to investigate the implications for the stability of sperm chromatin, which is essential for reproduction and, consequently, the survival of this species in the context of the environmental challenges facing the Mediterranean Sea. Further details on the effects of lead on the interaction of PL proteins with DNA were provided by computational analyses carried out in this study.

## 2. Materials and Methods

### 2.1. Ethics Statement

All experiments were conducted on *M. galloprovincialis* (Lamarck, 1819), which is not considered a protected species under Italian environmental legislation. This work was performed in accordance with the requirements of European legislation (Directive 2010/63) and Italian legislation (Legislative Decree 116/1992) on the ethical use and welfare of animals in research studies.

### 2.2. Lead Quantification in M. galloprovincialis Gonads

Nitric acid (70% *v*/*v*, analytical grade; VWR International S.r.l., Milano, Italy) and deionised water (18.2 MΩ·cm, produced in-house with a Sartorius arium^®^ pro system, Göttingen, Germany) were used throughout. Magnesium nitrate Mg(NO_3_)_2_ (1% Mg; AreaChem S.r.l., Napoli, Italy) and diammonium hydrogen phosphate (NH_4_)H_2_PO_4_ (PerkinElmer, Waltham, MA, USA) served as matrix modifiers. Working standard solutions of lead were prepared by serial dilution of a 1000 mg L^−1^ stock (Merck, Darmstadt, Germany) in 0.5 M HNO_3_. For sample preparation, approximately 0.050 g (±0.001 g) of each sample was weighed into a 15 mL glass tube with 3.0 mL of 70% nitric acid and 1.0 mL of deionised water. Sealed tubes were digested in a Milestone UltraWave microwave system (FKV S.r.l., Torre Boldone, Italy) running a two-step programme: ramp from 25 °C to 240 °C over 20 min at 1500 W, then a 10 min hold at 240 °C. Once cooled, digests were transferred to volumetric flasks and brought to 15 mL with deionised water. Total lead content was then measured by graphite furnace atomic absorption spectrometry (GF-AAS) on a PerkinElmer AAnalyst 800, equipped with Zeeman-effect background correction and an AS 800 autosampler. Absorbance was recorded at λ = 283.3 nm using a hollow cathode lamp, and concentrations were derived from external calibration curves. Atomisation was carried out at 1600 °C, preceded by drying at 110–130 °C, with argon as purge gas at 250 mL/min.

### 2.3. Mussels Sampling and Pb Exposure

The study used adult *M. galloprovincialis* mussels of mixed sex. These were collected in March 2025 and had an average shell length of 4.93 ± 0.17 cm. The specimens were provided by Eurofish Napoli S.R.L. in Baia, Naples. Fifteen mussels of unknown sex were exposed for 24 h at 18 ± 1 °C in laboratory tanks (36 × 22 × 22 cm) containing 7 L of 33% artificial seawater (ASW) with the following composition per litre: NaCl 29.2 g, KCl 0.60 g, MgCl_2_ 1.2 g, NaHCO_3_ 0.20 g, and CaCl_2_ 1.08 g as described in Marinaro et al. 2023 [52]. Specifically, the mussels were exposed to increasing concentrations of Pb: 0.5 µg/L PbCl_2_, 1.5 µg/L PbCl_2_, and 5 µg/L PbCl_2_. Meanwhile, 15 unexposed mussels were kept in a tank containing only ASW under the same temperature and exposure conditions.

### 2.4. Spermatozoa Sampling and Processing

The conditions of spermatozoa sampling and processing were performed following the protocols described in Marinaro et al. 2024 [53]. After 24 h of exposure, the mussels were carefully opened with a knife, making sure that the soft tissues stayed undamaged. The gonads were then placed in 500 μL of ASW at 16 °C for 5 min to stimulate gamete release. The gametes were observed under a microscope at 40× magnification to determine their sex. After sex identification, a section of the male gonads was used for further analysis. The ASW containing the spermatozoa was initially centrifuged at 2000× *g* for 2 min to discard debris. A second centrifugation at 9000× *g* for 10 min was then conducted, after which the spermatozoa contained in the supernatant were then collected. Both the sperm pellets and male gonadal tissues were stored at −80 °C.

### 2.5. Extraction of PL Proteins from M. galloprovincialis Spermatozoa

PLs extraction from both unexposed and Pb-exposed mussels was carried out using perchloric acid (5% final concentration), following the protocol reported in Marinaro et al. 2025 [54]. For each condition, N = 2 pellets of spermatozoa (prepared as specified above) were individually homogenised in 1 mL of distilled water with the use of a polypropylene pestle in a test tube, after which perchloric acid was added. Acid extraction was carried out with constant stirring overnight. The following day, the suspension was centrifuged at 13,000× *g* for 30 min at 4 °C, obtaining a supernatant containing only PLs and a pellet composed of cell debris. To remove residual perchloric acid, the supernatant was thoroughly dialyzed against distilled water. The proteins obtained were quantified using the Bradford assay [55].

### 2.6. Electrophoretic Analysis of PLs

The PL patterns were analyzed using two electrophoretic techniques: AU-PAGE, according to Marinaro et al. 2024 [22] and SDS-PAGE. For AU-PAGE, gels were prepared at 14% (*w*/*v*) with an acrylamide:bisacrylamide ratio of 25:0.67. Samples were denatured in 2.5 M urea for 30 min at room temperature before electrophoresis to ensure proper protein unfolding. Following sample preparation, 1 μL of acetic acid and pyronin was added, and electrophoresis was carried out for 1 h at 120 V using a 5% acetic acid running buffer. SDS-PAGE was carried out using a gel electrophoresis on 12.8% polyacrylamide gel in 0.025 M Tris-0.192 M glycine-0.1% SDS buffer, pH 8.8 at 25 mA. Afterwards, the gels were stained with Coomassie Brilliant Blue and images were obtained using a Gel-Doc system with ImageLab 6.0.1 (build 34) software (Bio-Rad, Hercules, CA, USA).

### 2.7. Plasmid DNA Preparation

High amounts of supercoiled pDNA were obtained by extracting the pGEM3 plasmid (2867 bp) from transformed *E. coli* HB101 cells with the ZymoPURE II Plasmid Midiprep Kit, following the precautions outlined in Carbone et al. 2012 [56]. Plasmid DNA was examined by gel electrophoresis using 1% agarose gels in TBE buffer solution (89 mM Tris-HCl, 2 mM EDTA and 89 mM boric acid at pH 8.0). The circular form of the pGEM3 plasmid was used for electrophoretic mobility shift assay (EMSA) and to assess the capacity of PLs to protect DNA.

### 2.8. Analysis of the Effect of M. galloprovincialis PL Proteins on DNA Electrophoretic Mobility (EMSA)

The impact of PL proteins from *M. galloprovincialis* on DNA mobility was evaluated using Electrophoretic Mobility Shift Assays (EMSA). The procedure was followed as published in Ferrero et al. 2024 [57] with some modifications. Specifically, each sample contained a constant amount of plasmid DNA (pGEM3, 150 ng) and increasing amounts of PL to achieve protein/DNA weight/weight ratios as described in detail in the Results Section 3.2. The experiments aimed to determine the protein/DNA ratio required for DNA saturation, defined as the appearance of a single DNA band close to the well. The samples were prepared by sequentially adding ultrapure water (Milli-Q), DNA and proteins until a final volume of 30 µL was obtained. They were then incubated at room temperature for 5 min to allow DNA-protein interactions. Electrophoretic conditions and visualization of DNA were carried out as described in Fioretti et al. 2012 [58] with few modifications. In particular, before gel electrophoresis, 10× TBE was added to the samples to obtain a final concentration of 1×, after which the samples were loaded on a 1% agarose gel in 1× TBE. Electrophoresis was then performed at 100 V for 30 min. Gels were stained with Safe View™ classic. Images of the gels were acquired with a Bio-Rad Gel-Doc system using Quantity One v.4.4.0 (Bio-Rad, Hercules, CA, USA).

### 2.9. DNA Protection Analysis

The capacity of PLs, isolated from the spermatozoa of mussels exposed to the three doses of Pb, to protect DNA from oxidative damage was examined using pGEM3 plasmid DNA exposed to 30 µM H_2_O_2_ and 5 µM CuCl_2_ (to induce the Fenton reaction and generate DNA breakage). Specifically, for the protection assay, 150 ng of plasmid DNA (pGEM3) and protein/DNA wt/wt ratios in a range from 0.1 to 0.3 were applied. After pre-incubating the DNA with PLs at room temperature for 5 min, H_2_O_2_ and CuCl_2_ were added to the reaction mixtures. The samples were then incubated in the dark for 30 min in a Thermoblock set to 37 °C. Electrophoretic analysis of the samples was performed on 1% agarose gel at 100 V for 30 min in 1× TBE. Following electrophoresis, agarose gels were stained with Safe View™ classic to view DNA migration, and the images of the gels were captured using the GelDoc Biorad (Hercules, CA, USA).

### 2.10. Salt-Induced Release of M. galloprovincialis Sperm Nuclear Basic Protein

The salt-induced release of *M. galloprovincialis* sperm nuclear basic proteins (SNBPs) was carried out following the procedure described in Carbone et al., 2023 [59]. For each Pb exposure and control condition, sperm pellets of approximately 120 mg were collected. Each pellet was resuspended in 1 mL of solution 1, containing 0.15 M NaCl, 25 mM EDTA (pH 8), and 1 mM PMSF. After being mixed with Solution 1, the samples were centrifuged at 4 °C for 10 min at 1900× *g*. The resulting pellets were then resuspended in 1 mL of a second solution containing 0.25 M sucrose, 5 mM MgCl_2_, 10 mM Tris-HCl (pH 8), 1 mM PMSF, and 0.38% Triton X-100. The suspensions were kept on ice for 10 min and subsequently centrifuged again at 4 °C for 10 min at 1900× *g*. 1 mL of a solution containing 0.25 M sucrose, 5 mM MgCl_2_ and 1 mM PMSF was added to the resulting pellet. The resulting mixture was centrifuged at 4 °C for 10 min at 1900× *g*. This process was repeated once. The final pellet obtained corresponded to the isolated sperm nuclei. The salt-induced extraction of sperm nuclear basic proteins (SNBPs) was performed according to the method described by De Guglielmo et al. 2019 [60]. For this aim, 1 mL of NaCl solutions at increasing concentrations was added to the isolated sperm nuclei, using the following salt gradients: 0.65 M, 0.8 M, 1 M, 2 M, 3 M, and 4 M NaCl. The sperm nuclei were sequentially resuspended with 1 mL of each of the NaCl solutions previously described and incubated at 4 °C for 30 min with agitation, further followed by centrifugation at 13,000× *g* for 30 min. A final concentration of 0.2 M HCl was consequently added to the collected supernatants to obtain total extraction of the SNBPs. The samples were then incubated and kept under stirring for 16 h at 4 °C, then centrifuged for 30 min at 13,000× *g*. The supernatants resulting from this process were extensively dialyzed under distilled water. A 4 µg aliquot of protein from each sample was analyzed by AU-PAGE to evaluate the amount of protein released from sperm nuclei in response to the increasing NaCl concentrations. The resulting protein bands were quantified using ImageJ version 1.54d. The intensity of the gel bands was quantified via densitometric analysis using ImageJ 1.50d (Wayne Rasband, NIH, Bethesda, MD, USA).

### 2.11. Statistical Analyses

Data regarding the salt-induced release of proteins from sperm nuclei were analyzed using two-way repeated measures ANOVA with Geisser-Greenhouse correction, followed by Dunnett’s post hoc test for multiple comparisons versus the unexposed control group, to evaluate differences between the means of the experimental groups at each NaCl concentration. Each protamine-like protein (PL-II, PL-III, and PL-IV) was analyzed independently. All statistical processing was performed using GraphPad Prism 10 (version 10.6.1). Results are presented as mean ± standard deviation (SD), and statistical significance was established at *p* < 0.05 within a 95% confidence interval. In the Supplementary Materials, tables with multiple comparisons are reported. Lead bioaccumulation data in gonadal tissue were analyzed using one-way ANOVA followed by Dunnett’s post hoc test for multiple comparisons against the unexposed control group. All statistical processing was performed using GraphPad Prism 10 (version 10.6.1). Results are presented as mean ± standard deviation (SD), and statistical significance was established at *p* < 0.05 within a 95% confidence interval.

### 2.12. Computational Modeling of Protamine-like Proteins, DNA and Lead

Due to the lack of experimentally determined three-dimensional structures and primary amino acid sequences for PL-III from *Mytilus galloprovincialis*, the homologous PL-III protein from *Mytilus californianus* was used in this study. Although the primary sequence of *M. galloprovincialis* is not currently available, species within the genus Mytilus (including *M. californianus*, *M. galloprovincialis*, *M. edulis*, and *M. trossulus*) form a closely related clade of blue mussels characterized by conserved genomic features and shared evolutionary histories. Comparative analyses of available Mytilus protamine-like (PL) proteins indicate strong conservation of the basic DNA-binding region, supporting the use of *M. californianus* PL-III as a suitable structural surrogate [61,62]. Multiple sequence alignment was performed using ClustalW 2.1 [63], confirming the presence of a conserved region among *M. californianus*, *M. trossulus*, and the sperm-specific protein Phi-1 from *M. edulis* (Figure S1). The corresponding gene sequence (GenBank accession DQ305039.1) was translated into the amino acid sequence using the ExPASy Translate tool [64], which served as the starting point for subsequent computational analyses. Residues 100–201 of the translated PL-III sequence (5′→3′, frame 3) exhibited the highest density of arginine and lysine residues. This highly basic region, conserved among protamines and PL proteins, mediates strong electrostatic interactions with the DNA phosphate backbone. Therefore, this segment was selected for structural modeling to focus the simulations on the region most relevant for protein–DNA interactions and potential metal coordination.

Residues potentially involved in metal coordination are present in proximity to this conserved region. In particular, cysteine and histidine residues may form strong coordination interactions with Pb^2+^ ions, while acidic residues such as glutamate and aspartate may contribute to weaker electrostatic or coordination interactions [40]. Accordingly, the structural model of PL-III was generated using AlphaFold2 [65,66]. In the predicted structure, α-helical regions showed high confidence scores (pLDDT 85–90), whereas disordered loop regions displayed lower confidence values (pLDDT 50–70).

For PL-II, the primary sequence from *M. galloprovincialis* was also unavailable; therefore, the homologous sequence from *M. californianus* (UniProt accession P22974; H1L_MYTCA) was used [67]. No additional suitable homologous sequences were available to perform a reliable multiple sequence alignment. A three-dimensional model was thus generated using AlphaFold2 and refined following the same protocol adopted for PL-III. In this model, α-helical regions exhibited very high confidence (pLDDT > 90), whereas loop regions showed lower confidence values (pLDDT 50–70).

The resulting PL-II and PL-III models were subsequently refined and energy-minimized using Avogadro (version 1.2.0; http://avogadro.cc/, accessed on 14 February 2026) and UCSF Chimera [68] to remove steric clashes and optimize local geometry. Hydrogen atoms were added, and amino acid protonation states were assigned according to physiological pH conditions.

A 12-base-pair double-stranded DNA fragment (CGCGAATTCGCG) was constructed and geometrically optimized. This canonical B-form sequence is widely used in computational studies as a model system for generic protein–DNA interactions.

Protein–DNA complexes were subjected to energy minimization consisting of 250 steps of steepest descent followed by 750 steps of conjugate gradient minimization, using the AMBER force field for both protein and DNA components. Since classical biomolecular force fields do not explicitly parameterize heavy metal ions such as Pb^2+^, lead–protein–DNA interactions were treated qualitatively. Pb^2+^ was represented using its formal +2 charge and an idealized coordination geometry. Accordingly, the resulting models provide comparative and mechanistic insights into interaction patterns rather than quantitative estimates of binding energies.

Pb^2+^ ions were also treated as ligands in docking simulations, with the aim of exploring potential coordination environments rather than predicting precise binding affinities.

### 2.13. Refinement of PL-II and PL-III by Molecular Dynamics

The PL-II and PL-III modeled structures were further refined through two independent 25 ns molecular dynamics (MD) simulations performed using GROMACS via the WebGro online platform [69] to assess overall structural stability. These simulations were intended solely to relax the initial models and ensure their stability; no quantitative dynamical analyses were performed. The resulting MD-refined structures were then used as input models for subsequent docking and interaction-prediction studies. MD simulations employed the GROMOS96 43a1 force field, with systems solvated using the SPC water model. Periodic boundary conditions were applied, and counterions were added to neutralize each system.

### 2.14. Docking Simulations

Molecular docking was performed using HADDOCK3 according to Dominguez et al., 2003 [70]. The protocol involved three sequential steps: (1) rigid-body docking with random orientations and energy minimization, generating 1000 structures; (2) semi-flexible refinement via simulated annealing in torsion-angle space, producing 200 structures; and (3) final refinement in explicit solvent, also generating 200 structures. The lead ion (Pb^2+^) was manually parametrized and treated as a ligand in both the protein–ligand and protein–DNA docking setups. Active and passive residues were defined based on the basic DNA-binding regions of PL-II and PL-III, respectively. Resulting structures were clustered based on RMSD and common contacts, and each cluster was scored using the HADDOCK score, which combines electrostatic, van der Waals, desolvation, and buried surface area energy terms, together with ambiguous interaction restraint (AIR) contributions. The ten most populated and highest-scoring clusters were visually inspected to identify the most probable binding modes and interaction networks. For clarity and reproducibility, all key docking parameters, including force field, water model, ligand parametrization, docking stages, and scoring terms—are summarized in Supplementary Table S1. This includes the use of the AMBER99SB-ILDN force field for proteins and DNA, the TIP3P water model for explicit solvent refinement, and manual parametrization of Pb^2+^ ions. Clustering criteria, active and passive residue definitions, and HADDOCK scoring components are also detailed in Table S1 to allow replication of the docking simulations.

### 2.15. Structural Visualization and Image Editing

The top-ranked docking complexes obtained from HADDOCK3 were analyzed using the Protein–Ligand Interaction Profiler (PLIP) [71] and visualized with PyMOL (The PyMOL Molecular Graphics System, Version 3.0 Schrödinger, LLC, New York, NY, USA). Figures were further refined and edited using Canva, a graphic design and image editing software (https://www.canva.com/).

## 3. Results

### 3.1. Lead Bioaccumulation in the Gonads of M. galloprovincialis

**Figure 1:**
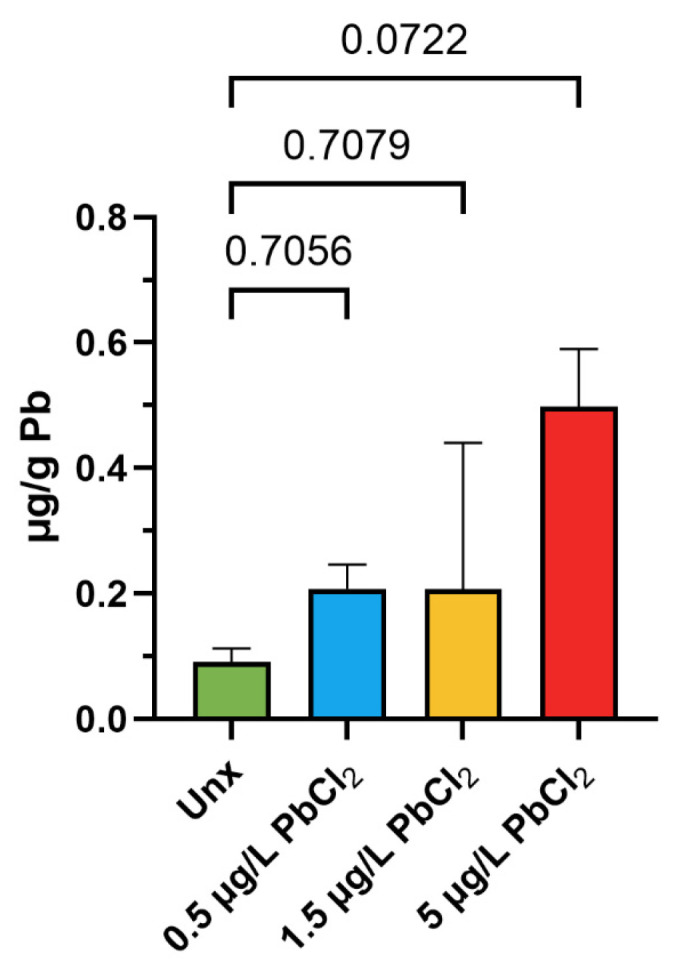
Lead (Pb) bioaccumulation in the gonads of *M. galloprovincialis*. Pb concentration (µg/g dry weight) measured in gonadal tissue of unexposed mussels (Unx) and mussels exposed to 0.5, 1.5, and 5 µg/L PbCl_2_ for 24 h. Data are expressed as mean ± SD. Statistical comparisons between the Unx group and each exposure condition, with the corresponding *p*-values reported above (*p* = 0.7056 for Unx vs. 0.5 µg/L; *p* = 0.7079 for Unx vs. 1.5 µg/L; *p* = 0.0722 for Unx vs. 5 µg/L). *n* = 2.

To verify that the experimental exposure conditions resulted in effective uptake of lead by the animals, the concentration of Pb in gonadal tissue was quantified in unexposed mussels and in animals exposed to 0.5, 1.5, and 5 µg/L PbCl_2_. As shown in Figure 1, the gonadal Pb content in unexposed mussels was approximately 0.10 µg/g dry weight. Following exposure to 0.5 and 1.5 µg/L PbCl_2_, the gonadal Pb concentrations increased to approximately 0.20 µg/g, representing a roughly two-fold elevation relative to the control. At the highest exposure dose (5 µg/L PbCl_2_), a further and more marked increase was observed, reaching approximately 0.49 µg/g, corresponding to an approximately five-fold increase compared to the unexposed group. Taken together, these data indicate a dose-dependent trend in Pb accumulation in the gonads.

### 3.2. Analyses of PLs by AU-PAGE and SDS-PAGE

**Figure 2:**
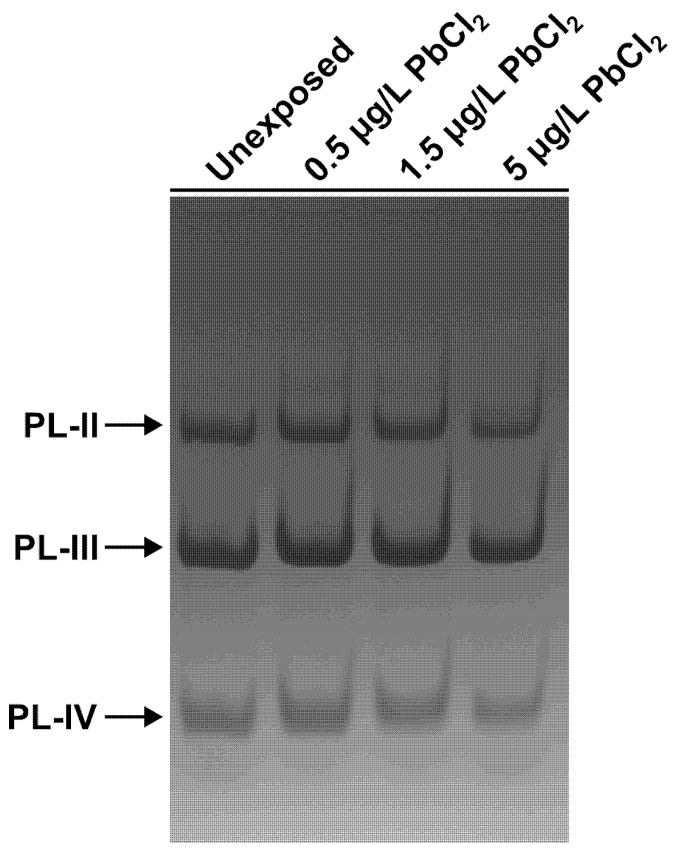
AU-PAGE of PLs extracted from: Unx mussels; mussels exposed to: 0.5 µg/L 1.5 µg/L and 5 µg/L PbCl_2_. *n* = 2.

**Figure 3:**
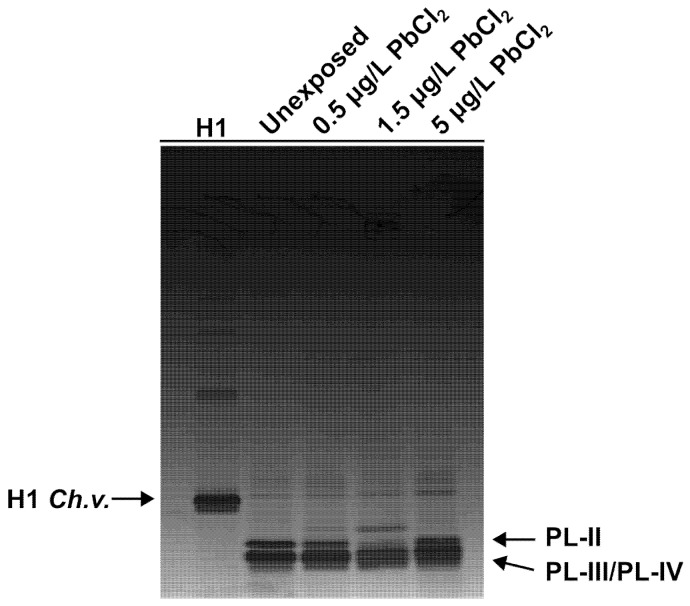
SDS-PAGE of PLs extracted from: Unx mussels; mussels exposed to: 0.5 µg/L 1.5 µg/L and 5 µg/L PbCl_2_. *n* = 2.

PLs extracted from unexposed and exposed mussels to different concentrations of PbCl_2_ were analyzed using two electrophoretic approaches: AU-PAGE and SDS-PAGE in order to assess potential alterations in the electrophoretic pattern induced by PbCl_2_ exposure. AU-PAGE analysis revealed three distinct protein bands corresponding to PL-II, PL-III and PL-IV, which migrated from top to bottom in all samples. A comparison of the electrophoretic profiles revealed no significant differences between the PLs extracted from control mussels and those isolated from mussels exposed to various concentrations of PbCl_2_. The same protein samples were also analyzed by SDS-PAGE. In control samples, three bands were observed in the expected order: PL-II, PL-III, and PL-IV, corresponding to their approximate molecular masses. As expected, PL-II and PL-III, which have very similar molecular weights (13.8 and 11.8kDa, respectively), displayed similar migration patterns. In contrast to the AU-PAGE results, SDS-PAGE revealed alterations in the electrophoretic pattern of PLs from mussels exposed to 1.5 µg/L and 5 µg/L PbCl_2_. These changes, particularly evident for PL-II and PL-III, suggest that Pb exposure may induce conformational changes that affect how these proteins interact with SDS and migrate in SDS-PAGE (Figure 2 and Figure 3).

### 3.3. Evaluation of the DNA–PLs Interaction by EMSA

**Figure 4:**
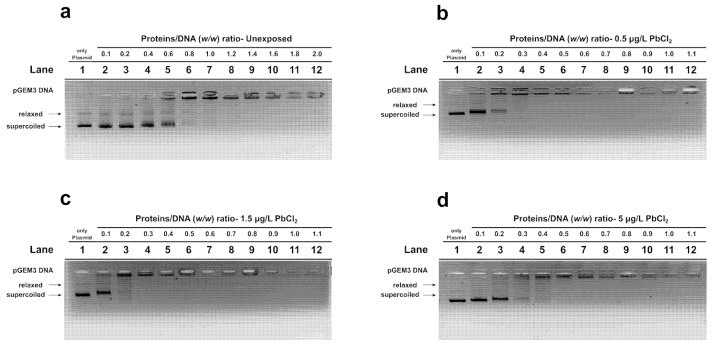
Analysis of the DNA-binding ability of PLs by EMSA (**a**) Unx mussels, mussels exposed to: (**b**) 0.5 µg/L PbCl_2_, (**c**) 1.5 µg/L PbCl_2_, (**d**) 5 µg/L PbCl_2_. *n* = 2.

EMSA was used to evaluate changes in the DNA-binding ability of PLs isolated from unexposed and exposed mussels to increasing concentrations of PbCl_2_. The amount of plasmid DNA was kept at 150 ng in all samples, while the protein concentration was increased step by step to obtain protein/DNA ratios (*w*/*w*) with the numbers indicated on the wells in the figures. DNA saturation was achieved in unexposed mussels at a ratio of approximately 1.4. In contrast, PLs from mussels exposed to all concentrations of PbCl_2_ produced DNA saturation at a lower protein/DNA ratio. Specifically, after exposure to 0.5 µg/L and 1.5 µg/L of PbCl_2_, DNA saturation was observed at protein/DNA ratios of 0.4 and 0.3, respectively. Following exposure to 5 µg/L of PbCl_2_, however, DNA saturation was obtained at a protein/DNA ratio of 0.5. Overall, these data showed that exposure to PbCl_2_ enhances the DNA-binding ability of PL proteins, particularly at low-to-intermediate exposure levels (Figure 4).

### 3.4. DNA Protection Assays

**Figure 5:**
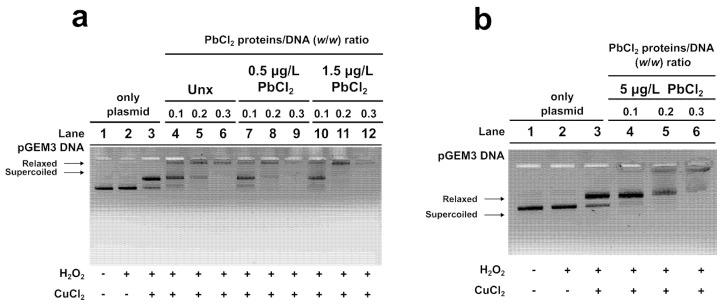
DNA protection assays of PLs extracted from Unx mussels (**a**) and mussels exposed to: 0.5 µg/L PbCl_2_ (**a**), 1.5 µg/L PbCl_2_ (**a**), 5 µg/L PbCl_2_ (**b**). *n* = 2.

The capacity of the PLs to protect DNA from oxidative damage was assessed through DNA protection assays. For these assays, samples containing a fixed quantity of plasmid DNA (150 ng) and increasing protein amounts corresponding to protein/DNA ratios between 0.1, 0.2, and 0.3 were prepared. Oxidative stress was induced through the addition of H_2_O_2_ and CuCl_2_ to trigger the Fenton reaction, generating reactive radicals that convert a fraction of supercoiled DNA into its relaxed form. As expected, in plasmid DNA samples treated only with H_2_O_2_ and CuCl_2_, the majority of the circular plasmid DNA was in relaxed form, confirming the induction of oxidative damage. The presence of PLs altered this pattern depending on their source. The DNA was fully protected at a protein/DNA ratio of 0.3 by PLs from unexposed mussels, with the relaxed and supercoiled DNA bands completely absent. Similarly, PLs from mussels exposed to 0.5 µg/L PbCl_2_ also provided complete protection at a ratio of 0.3. Remarkably, PLs from the mussels’ exposure to 1.5 µg/L PbCl_2_ protected DNA even more efficiently, achieving near-complete protection already at a ratio protein/DNA of 0.2. In contrast, PLs from mussels exposed to 5 µg/L PbCl_2_ exhibited reduced protective capacity, as evidenced by a persistent relaxed DNA band and incomplete saturation even at the highest tested ratio (0.3). These results suggest that moderate Pb exposure may alter both DNA binding ability and DNA-protective function of PLs, whereas higher Pb concentrations may impair their ability to safeguard DNA from oxidative damage (Figure 5).

### 3.5. Salt-Induced Extraction of PLs

**Figure 6:**
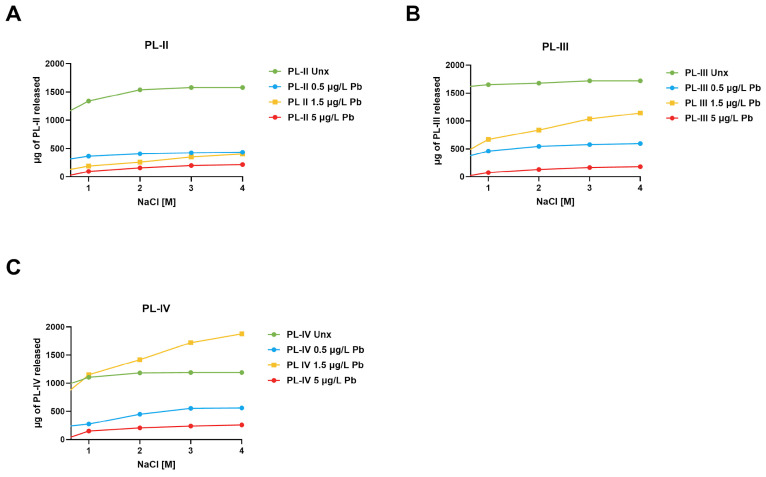
Release of PLs from sperm nuclei at different NaCl molar concentrations. Micrograms of PL-II (**A**), PL-III (**B**) and PL-IV (**C**) released from sperm nuclei with increasing molar NaCl concentration in different exposure conditions. *n* = 2.

To determine whether Pb exposure affects the association of PL proteins with sperm DNA, sperm nuclei were isolated from mussels exposed or not to PbCl_2_, and SNBP was extracted by increasing NaCl concentrations (0.5–4 M). In unexposed mussels, PL-II showed increasing release with higher NaCl concentrations, reaching high levels (approximately 1500–1600 μg) at 3–4 M NaCl. By contrast, exposure to all concentrations of PbCl_2_ significantly reduced the amount of PL-II. The most marked reduction was observed at a concentration of 5 µg/L of PbCl_2_, where the release of PL-II reached the minimum level. A similar trend was observed for PL-III, for which extractable levels also decreased in mussels exposed to PbCl_2_. In this case, the reduction in release of this protein was smaller at 0.5 and 1.5 μg/L compared to PL-II, but the highest dose (5 μg/L) again showed the lowest release (Figure 6).

### 3.6. Computational Modeling of PL-II and PL-III

**Figure 7:**
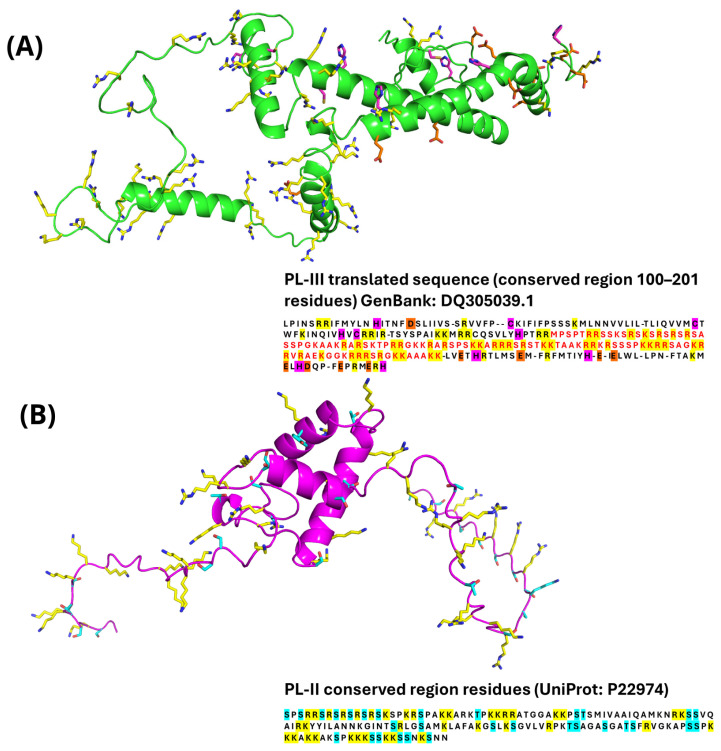
(**A**) Modeled structure of the PL-III sequence from *Mytilus californianus* (shown as a green cartoon), predicted by AlphaFold2 and further refined through molecular dynamics (MD) simulations. Residues in conserved regions are labeled in red. High-affinity coordination residues, cysteines and histidines, are highlighted in magenta, medium-affinity acidic residues, glutamates and aspartates, in orange, and positively charged residues, arginines and lysines, in yellow. (**B**) Modeled structure of the PL-II sequence from *Mytilus californianus* (magenta cartoon), also predicted using AlphaFold2 and refined with MD simulations. Polar residues (serines and threonines) indicated as potential weak Pb^2+^ coordination sites are in cyano and positively charged residues, arginines and lysines are in yellow for both proteins; the corresponding primary amino acid sequences are colored by atom type.

To investigate the potential molecular mechanisms of lead binding to mussel PL-II and PL-III and its possible impact on DNA interaction, we performed computational analyses. For PL-III from *M. galloprovincialis*, no experimental structure or primary amino-acid sequence is available in public databases. Therefore, the conserved region, rich in basic residues potentially involved in DNA-binding, was modeled using the homologous sequence from *Mytilus californianus* (PL-III), and the region corresponding to residues 100–201 was selected based on its high content of arginine and lysine residues. This segment is conserved among protamines and PL proteins and is known to mediate strong electrostatic interactions with the DNA phosphate backbone. Sequence analysis identified cysteine and histidine residues arranged in a spatially favorable configuration near the conserved region, which may serve as high-affinity coordination sites for Pb^2+^. Acidic residues such as glutamate and aspartate may also contribute to weaker coordination interactions, while the abundant arginine and lysine residues likely facilitate electrostatic attraction of the metal toward the DNA-binding region [55]. These features suggest a synergistic mechanism in which high- and moderate-affinity residues cooperate to modulate Pb–PL-III interaction and potentially affect DNA binding (Figure 7A). For PL-II, the homologous DNA-binding sequence from *Mytilus californianus* (UniProt P22974; H1L_MYTCA) was used. The PL-II domain is rich in basic residues (Figure 7B), which favor DNA interaction, and contains polar residues such as serine, threonine, and tyrosine. Although these residues can form hydrogen bonds with DNA, they are expected to have limited metal-coordination capacity compared to cysteine, histidine, aspartate, and glutamate [55]. From a structural perspective, PL-II resembles histone-like proteins, with a DNA-binding domain organization that supports electrostatic stabilization of nucleoprotein architecture. Interaction analysis suggests the formation of weak coordination interactions, mainly involving polar residues, indicating a lower propensity of PL-II to establish stable Pb^2+^ coordination sites compared to PL-III.

### 3.7. Computer-Aided Molecular Docking Studies

The binding modes of PL-II and PL-III with DNA and Pb^2+^ ions were investigated using molecular docking simulations performed by HADDOCK3. The analysis aimed to identify preferred binding modes and assess whether Pb^2+^ can directly interact with PL-II, PL-III, and DNA, potentially affecting their structural stability and biological function.

**Figure 8:**
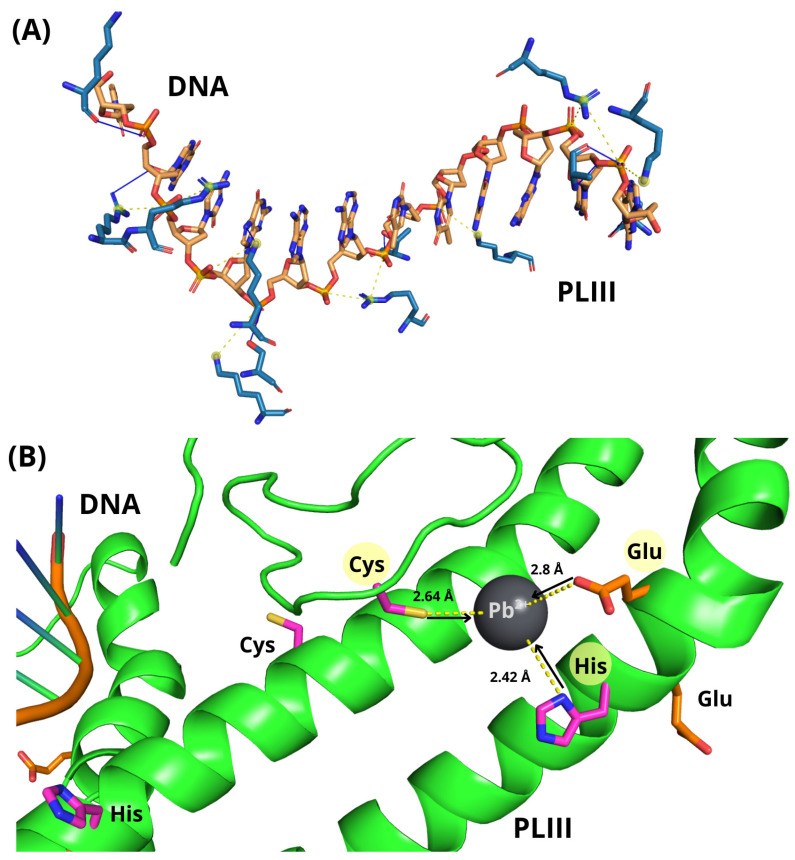
Docked models of the top-ranked complexes between PL-III and DNA (**A**) and the PL-III/Pb^2+^ complex (**B**). (**A**) Only PL-III residues directly involved in DNA binding are shown as blue sticks for clarity. DNA strands are represented as orange sticks, with atoms colored by element. Hydrogen bonds are depicted as solid blue lines and salt bridges as yellow dashed lines. (**B**) PL-III is shown as a green cartoon. Key residues within the DNA-binding domain are highlighted: cysteines and histidines in magenta, and glutamates in orange. Metal–ligand coordination is indicated by black arrows pointing from electron-donor atoms to the Pb^2+^ ion, while yellow dashed lines denote Pb^2+^–S, Pb^2+^–N, and Pb^2+^–O bond distances. Residues involved in coordination are highlighted in yellow.

**Figure 9:**
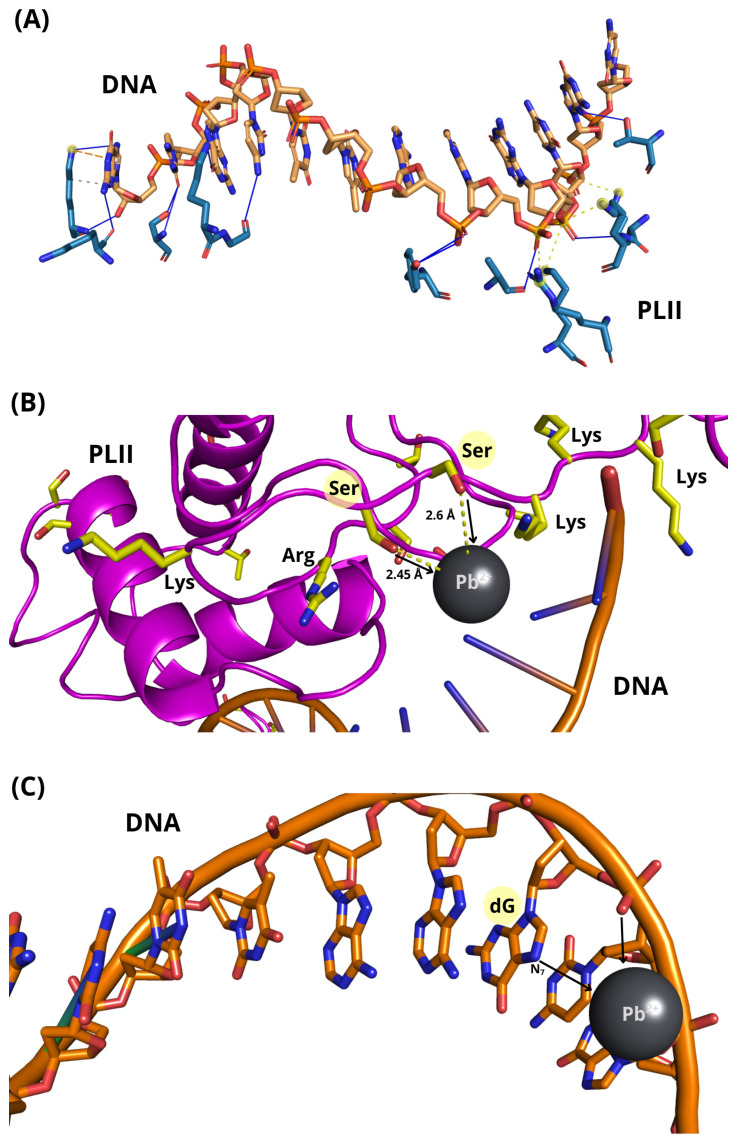
Docked models of the top-ranked complexes between PL-II and DNA (**A**), the PL-II/Pb^2+^ complex (**B**), and the DNA/Pb^2+^ complex (**C**). (**A**) Only PL-II residues directly involved in DNA binding are shown as blue sticks for clarity. DNA strands are represented as orange sticks, with atoms colored by element. Hydrogen bonds are depicted as solid blue lines, salt bridges as yellow dashed lines, and the cation–π interaction as an orange dashed line. (**B**) PL-II is shown as a magenta cartoon, with key residues of the DNA-binding domain highlighted as yellow sticks. Metal–ligand coordination is indicated by black arrows pointing from donor oxygen atoms (serine hydroxyl groups) to the Pb^2+^ ion, while yellow dashed lines denote Pb^2+^–O bond distances. (**C**) Pb^2+^ coordination to DNA involves the N_7_ position of guanine bases and oxygen atoms of the phosphate backbone. DNA is represented as orange sticks, and Pb^2+^ as a dark gray sphere. Residues and nucleotides involved in coordination are highlighted in yellow.

Docking of the modeled structure of PL-III with DNA (top-ranking cluster: HADDOCK score = −55.2 ± 2.5) revealed extensive electrostatic interactions, primarily involving lysine and arginine side chains and backbone atoms with the DNA phosphate groups. These interactions consisted mainly of salt bridges and hydrogen bonds, indicating strong electrostatic complementarity and contributing to the stability of the PL-III/DNA complex (Figure 8A). Lead-binding simulations for PL-III (lowest-energy cluster: HADDOCK score = −48.2 ± 6.2) indicated a favorable coordination geometry within the DNA-binding domain (Figure 8B). Cysteine and histidine residues were predicted to act as primary ligands, forming strong coordination interactions with Pb^2+^, while acidic residues such as glutamates were predicted to contribute to coordination and electrostatic stabilization. The predicted coordination distances (Pb–S ≈ 2.6 Å, Pb–N ≈ 2.4 Å, Pb–O ≈ 2.8 Å) are consistent with previously reported Pb^2+^ coordination geometries [72]. Several residues involved in Pb^2+^ coordination spatially overlapped with those implicated in DNA binding, suggesting that lead binding may compete with and perturb the PL-III–DNA interface, potentially altering the electrostatic network stabilizing the complex. Docking simulations of the modeled structure of PL-II with DNA (best-scoring cluster: HADDOCK score = −58.6 ± 3.4) revealed a similar interaction network (Figure 9A). Stabilization of the complex was mainly driven by salt bridges with the DNA phosphate backbone, along with hydrogen bonds involving purine base nitrogen atoms and protein backbone/side-chain atoms. Additional stabilization was provided by a cation–π interaction between a lysine side chain and the aromatic ring of deoxyguanosine, suggesting that PL-II/DNA binding is governed by electrostatic forces combined with non-covalent aromatic interactions.

For the PL-II/Pb^2+^ complex (best-scoring cluster: HADDOCK score = −46.5 ± 4.3), the lead ion was predicted to coordinate primarily through hydroxyl oxygen atoms of serine residues within the DNA-binding domain, with Pb–O distances of approximately 2.6 Å (Figure 9B). Although coordination via serine hydroxyl groups is generally weaker than that mediated by cysteine or histidine, the local enrichment of lysine and arginine residues may generate a positively charged electrostatic environment that facilitates Pb^2+^ retention near the coordinating residues, possibly favoring transient interactions with other polar residues. Docking of Pb^2+^ with DNA (best-scoring cluster: HADDOCK score = −60.3 ± 5.4) indicated a stable coordination complex in which Pb^2+^ interacts with the N_7_ position of deoxyguanosine and an oxygen atom of the phosphate backbone, consistent with previously reported DNA–Pb^2+^ coordination modes [73] (Figure 9C). This dual coordination highlights the ability of Pb^2+^ to interact with multiple electron-donating sites within DNA, potentially affecting its local structure and electronic properties.

**Figure 10:**
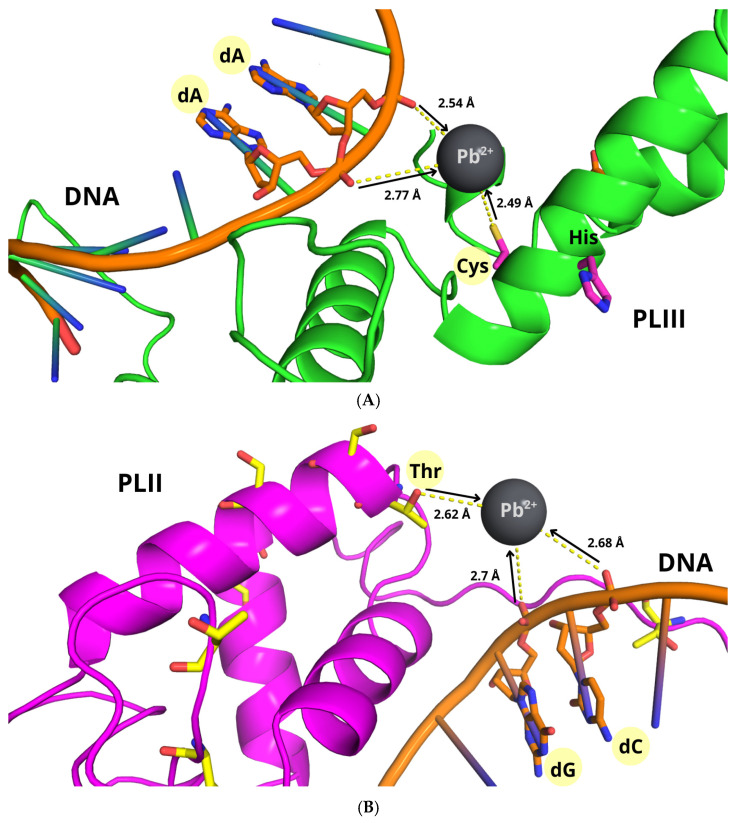
Docked models of the top-ranked ternary complexes PL-III/Pb^2+^/DNA (**A**) and PL-II/Pb^2+^/DNA (**B**). PL-III and PL-II are depicted as green and magenta cartoons, respectively. Metal–ligand coordination is illustrated by black arrows pointing from donor atoms to the Pb^2+^ ion, while yellow dashed lines indicate Pb^2+^–O and Pb^2+^–S bond distances. DNA is shown as orange sticks, and the Pb^2+^ ion as a dark gray sphere. Residues and nucleotides involved in coordination are highlighted in yellow.

To further investigate whether lead could compete with DNA for binding to PL proteins, we extended our docking analysis to include ternary PL-II/PL-III–DNA–Pb models. This approach allows the simultaneous evaluation of protein–DNA, protein–metal, and DNA–metal interactions, providing a more comprehensive view of potential competitive and cooperative binding mechanisms. In the best clustered solution obtained with HADDOCK (HADDOCK score = −56 ± 2.4) for the PL-III/DNA/Pb complex, Pb^2+^ could display a strong propensity for multivalent coordination. The metal ion could form coordination bonds with a high-affinity residue such as cysteine, while also interacting with the phosphate oxygen atoms of the DNA backbone (Figure 9A). This configuration could suggest that Pb^2+^ might function as a bridge between the protein and DNA, potentially contributing to the stabilization of the ternary complex. By contrast, in the PL-II/DNA/Pb complex (HADDOCK score = −51 ± 3.6), the coordination network could be less defined and overall weaker. In this case, Pb^2+^ could interact with a threonine residue, considered a medium-affinity coordinating site, and maintain contact with the phosphate backbone oxygens of guanine and cytosine nucleotides (Figure 9B). The lower strength and specificity of these interactions could be consistent with a reduced stabilizing effect compared to the PL-III system. Taken together, these results could suggest that Pb^2+^ might not simply compete with DNA for binding to PL. Rather, it could also facilitate the formation of ternary complexes through bridging interactions, with coordination patterns that could vary depending on the protein isoform. This could support a more nuanced model in which lead could modulate PL–DNA recognition through both competitive and cooperative structural effects (Figure 10).

## 4. Discussion

Lead (Pb) is a ubiquitous environmental contaminant that remains a significant risk to ecosystems and living organisms, regardless of regulatory efforts to limit its use [61,62,74,75,76]. Due to its non-biodegradable nature and long environmental persistence, lead remains a contaminant of major concern in both terrestrial and aquatic ecosystems. Several studies have demonstrated that this contaminant easily accumulates in biological tissues such as the liver and kidneys, where it interferes with essential physiological and biochemical processes [64,65,66,67,68,77]. Chronic exposure has been associated with neurotoxicity and oxidative stress. Among the biological systems most sensitive to lead toxicity, the reproductive system has emerged as a particular target. A growing number of epidemiological and experimental studies suggest that lead exposure negatively affects male reproductive health. In humans and animal models, elevated levels of lead in blood or tissues have been linked to reduced sperm quality, including decreased concentration, motility, and normal morphology of spermatozoa, thereby compromising fertility [78,79,80]. In this context, growing interest has been focused on investigating biological responses to lead exposure at multiple levels of biological organization, from whole-organism effects to molecular and biochemical alterations. On the other hand, the effect of epigenetic changes on how organisms react to various types of stress is well understood [81]. Lead effects on the male reproductive system and associated molecular mechanisms are not yet fully elucidated. The use of bioindicator organisms, such as marine bivalve molluscs, and in particular *M. galloprovincialis*, has proven to be an effective strategy for assessing environmental pollution. The present study aims to clarify the molecular effects of lead exposure on the reproductive health of the bivalve mollusc *M. galloprovincialis*. The study focuses on the impact of lead on protamine-like (PL) proteins. Using a combination of electrophoretic, biochemical and computational approaches, the study assesses whether exposure to three different concentrations of PbCl_2_ (0.5 µg/L, 1.5 µg/L and 5 µg/L) results in changes to the structure and function of PL proteins through direct metal-protein interactions. Although the concentration of free Pb^2+^ may be lower than that of other chemical species present in the environment (e.g., PbCl^+^, PbCl_2_, PbCl_3_^+^), Pb^2+^ is the ionic form primarily responsible for the observed toxic effects [37]. To investigate how exposure to PbCl_2_ affects the interaction between protamine-like (PL) proteins and DNA in *M. galloprovincialis*, a series of biochemical assays were performed, including Electrophoretic Mobility Shift Assay (EMSA), DNA protection assays against oxidative damage, and salt-induced extraction of nuclear basic proteins from sperm nuclei. By AU-PAGE, the absence of mobility shifts suggested that Pb exposure does not markedly affect the net charge or charge/mass ratio of these proteins under the AU-PAGE conditions. The same protein samples, analyzed by SDS-PAGE to evaluate potential changes in protein size or conformation under denaturing conditions, showed alterations, particularly evident for PL-III. These deviations suggest that PbCl_2_ exposure may induce conformational changes, partial unfolding, or post-translational modifications that affect how the protein interacts with SDS and migrates in the denaturing gel. The apparent discrepancy between AU-PAGE and SDS-PAGE results can be explained by the fundamentally different separation principles underlying these two electrophoretic techniques. In SDS-PAGE, the protein is denatured. SDS binds along the chain, imparting a uniform negative charge proportional to the mass. Therefore, migration depends almost entirely on size (i.e., molecular weight). If a metal ion (e.g., Zn^2+^, Cu^2+^ or Ba^2+^) is present, induces cross-linking (bridges between proteins), stabilizes structures that are resistant to denaturation, and causes aggregation or covalent modification, then the apparent mass or shape changes and broader bands (oligomers) could be visible, as well as smeared or altered mobility. These effects become very visible because SDS eliminates the charge variable and highlights any structural or mass changes. AU-PAGE, instead, separates proteins based on their charge-to-mass ratio and is commonly used to evaluate the protein pattern of extremely basic proteins such as protamine-like (PL) proteins and protamines. In AU-PAGE the protein retains its actual charge. Migration depends on charge-to-mass ratio. Metals often alter local charges, induce small conformational changes, and bind reversibly. However, these variations may cancel each other out, mass and charge both increase, shape changes that do not drastically alter mobility. In addition, our findings indicated that lead exposure induces dose-dependent alterations in protamine-like (PL) protein function in *M. galloprovincialis*. At all concentrations of PbCl_2_, PLs exhibited an ‘all or nothing’ binding mode to DNA [82]. However, early DNA saturation was observed, which indicates altered DNA binding, suggesting abnormal chromatin compaction. This is in line with the findings that lead interaction with HP2 caused a dose-dependent decrease in HP2 binding to DNA, suggesting that lead may alter chromatin stability [83]. In addition, in mice, it has been reported that lead accumulation into the nucleus and Pb binding to nuclear sulfhydryl groups decreased chromatin decondensation in vitro [84]. Moreover, at the highest concentrations tested (5 µg/L PbCl_2_), the protective capacity of PL proteins against oxidative damage was impaired, leaving DNA more vulnerable to strand breaks.

These findings are consistent with the study by Li et al. [85], which demonstrated that lead exposure promotes the generation of reactive oxygen species (ROS) in semen. Elevated ROS levels in spermatozoa can damage DNA, leading to increased fragmentation [80]. In this regard, the discrepancies observed between the EMSA and protection assay results can be explained by distinguishing between DNA-binding affinity and the functional quality of protein-DNA interactions. Exposure to lead (PbCl_2_) appears to increase the binding affinity of PL proteins to DNA by coordinating with cysteine and histidine residues. This could create rigid molecular bridges that stabilise the complex in a non-physiological manner. This increases binding viscosity without necessarily improving function. At lower concentrations, PL proteins retain sufficient protective capacity despite altered binding dynamics. However, at the highest concentration (5 µg/L PbCl_2_), a toxic threshold appears to be reached where the quantitative reduction in PL-II and PL-III proteins, combined with conformational alterations, could generate defective chromatin structures. In line with these observations, we detected altered electrophoretic mobility of PL proteins and reduced extractability from sperm nuclei. Collectively, these results suggest that lead induces direct metal–protein interactions that may cause conformational perturbations and impair protein–DNA interactions. Computational modeling further supports these experimental observations, indicating that PL-III is particularly susceptible to Pb interference due to the presence of cysteine and histidine residues capable of forming high-affinity coordination sites. Importantly, these metal-binding residues overlap with key DNA-binding regions, suggesting that lead may compete directly with DNA for binding to PL-III, thereby destabilizing the PL-III/DNA complex and potentially disrupting sperm chromatin organization. Although molecular docking represents a simplified model compared with the highly compact and dynamic organization of sperm chromatin, the simulations performed in this study were conducted under physiological conditions to approximate the biochemical environment of PL–DNA interactions. To further investigate the potential role of lead in modulating these interactions, we implemented a ternary docking model including protamine-like proteins, Pb, and DNA. This approach enabled the identification of potential sites where lead could interfere with PL–DNA binding, providing mechanistic insight into how Pb may affect the stability and configuration of the nucleoprotein complex. Importantly, these computational observations are consistent with our in vitro EMSA results, supporting the interpretation that lead can perturb PL–DNA interactions. The docking simulations suggest that Pb^2+^ may bind within the DNA minor groove, potentially competing with PL proteins. However, the EMSA experiments do not conclusively demonstrate that protein displacement occurs through direct site competition. Alternatively, Pb may induce changes in DNA curvature or flexibility, indirectly affecting PL–DNA binding. Therefore, although the combined computational and experimental analyses support a Pb-induced perturbation of the nucleoprotein complex, further high-resolution structural studies will be required to distinguish between direct competition and indirect DNA-mediated effects.

This observation aligns with evidence that lead is capable of interacting with HP2 [86]. This modification affects the binding of DNA–protamine, potentially leading to alterations in chromatin structure, which may contribute to male fertility problems and possibly cause DNA damage. Although in this study, the effect of lead chloride on individual protamine-like proteins has not been determined, it would appear that the most vulnerable protein to the effects of lead is PL-III, as indicated by molecular docking. However, our previous work on exposure to other heavy metals suggests that, generally, PL-II is more vulnerable, due to its more peripheral location in the sperm chromatin of this organism [53,54,87]. In this case, PL-II lacks strong metal-binding ligands and is predicted to coordinate Pb^2+^ only weakly via serine residues, implying a lower propensity for stable lead binding; however, transient interactions and electrostatic perturbations cannot be excluded. Moreover, Pb^2+^ may directly interact with DNA through coordination at guanine N_7_ and phosphate oxygen atoms, indicating a dual mechanism by which lead can compromise nucleoprotein stability. In this bioindicator species, the results suggest a potentially conserved mechanism of lead toxicity that may also extend to mammals, as human protamines contain metal-binding sites and could undergo similar structural and functional disruptions upon Pb^2+^ exposure, potentially compromising male fertility. Unlike Hg(II) and Cr(VI), which cause co-migration of PL-II and PL-III through strong metal–thiolate and metal–nitrogen interactions, Pb(II) affects both proteins as distinguishable bands, consistent with its HSAB classification as a borderline-to-soft acid that forms defined coordination complexes with cysteine, histidine, and serine residues rather than targeting sulfhydryl groups indiscriminately [88]. Pb(II) also differs in environmental behavior: unlike Hg and Cd, it adsorbs strongly onto particles and sediments, producing episodic bioavailability [89,90], yet our data show it can impair reproductive protein integrity at environmentally relevant doses, potentially below thresholds of somatic toxicity [22,52,91]. The broader significance of these findings is supported by studies on human protamine HP2, where Pb^2+^ competes with Zn^2+^ and reduces HP2–DNA binding dose-dependently, pointing to a conserved pathway linking heavy metal exposure to reproductive dysfunction across phylogenetically distant species [22,52,92,93]. Although the present study focused on Pb(II) to investigate its specific effects on PL proteins and sperm chromatin of *M. galloprovincialis*, it is important to acknowledge that in marine environments lead rarely occurs in isolation, as it frequently coexists with other heavy metals such as cadmium (Cd), mercury (Hg), copper (Cu), and arsenic (As) as a consequence of industrial, mining, and agricultural runoff. Co-occurring metals are well documented to display synergistic toxicity, whereby the combined effect exceeds the sum of individual impacts [94,95,96,97]. Lead (Pb) in particular acts as a toxic intensifier, exacerbating oxidative stress, cellular damage, and physiological impairment induced by co-occurring metals even at low environmental concentrations [98]. Among the most relevant binary interactions, Pb combined with Hg enhances neurotoxicity through disruption of neurotransmission and synaptic homeostasis, producing stronger neurobehavioral effects than either metal alone, while Pb combined with Cd increases oxidative stress and histological damage to gills and liver, often reducing population density in aquatic invertebrates and fish. Similarly, co-exposure to Pb and As results in more pronounced brain damage and impaired swimming behavior in fish, attributable to interference with neurotransmitter signaling and HPI axis function, whereas Pb and Cu mutually facilitate uptake by gills and tissues, thereby increasing bioaccumulation and overall toxicity [99,100]. The underlying mechanisms driving these synergistic effects include elevated reactive oxygen species (ROS) production, inhibition of antioxidant defenses such as superoxide dismutase (SOD) and glutathione S-transferase (GST), impaired neurotransmission, enhanced bioaccumulation, and the overwhelming of detoxification systems including metallothioneins [94,101]. From an environmental risk perspective, these findings imply that safety thresholds based solely on single-metal toxicity data are likely to underestimate actual hazards, since co-exposure typically results in higher metal accumulation in gills, kidney, and liver; increased organ degeneration; reduced reproductive success; and developmental abnormalities in offspring. Notably, the combination of Pb, Cd, and Hg has been referred to as a “toxic trio” owing to its particularly severe impact on marine organisms—including mussels, fish, and crabs—with potential consequences for human health through trophic biomagnification. In this study, we observed an accumulation of Pb in the gonads, with the highest accumulation occurring at an exposure dose of 5 µg/L. At this exposure dose, we also observed significant alterations in the protective function of PL proteins on DNA in prooxidant conditions, indicative of a different binding to DNA, also confirmed by a notable reduction in the release of these proteins from sperm nuclei.

## 5. Conclusions

In conclusion, the present study provides evidence that lead exposure may contribute to reproductive toxicity in the bioindicator species *M. galloprovincialis* through alterations at the molecular level. PbCl_2_ exposure was associated with structural and functional changes in PL proteins, with PL-III appearing particularly affected. Electrophoretic analyses, supported by computational modeling, suggest that lead may interact with cysteine and histidine residues located within DNA-binding domains, potentially inducing conformational changes that could affect the stability of the nucleoprotein complex. The observed shifts in DNA saturation profiles and the partial impairment of the DNA-protective capacity of PL proteins at the highest exposure dose are consistent with a scenario in which exposure may predispose spermatozoa to altered chromatin condensation and increased susceptibility to DNA damage. Overall, this study contributes to the growing body of evidence linking environmental lead contamination to reproductive effects at the sperm chromatin level and highlights the importance of *M. galloprovincialis* as a model for studying the molecular basis of metal toxicity in marine ecosystems. Given the structural conservation of basic nuclear proteins across metazoans, these findings raise the possibility of shared mechanistic features with heavy-metal-induced reproductive impairment in other organisms; however, direct extrapolation to vertebrates, including humans, requires further experimental validation.

## Data Availability

The original contributions presented in this study are included in the article/Supplementary Materials. Further inquiries can be directed to the corresponding author.

## Supplementary Materials

The following supporting information can be downloaded at: https://www.mdpi.com/article/10.3390/biom16040529/s1, Figure S1: Multiple sequence alignment of PL-III proteins from *Mytilus* species. Table S1. Key parameters used in the HADDOCK3 molecular docking simulations. Table S2: Dunnett’s multiple comparisons test following one-way ANOVA of Pb gonadal content (original data for Figure 1). Table S3: Dunnett’s multiple comparisons test following one-way ANOVA of Pb gonadal content, PL-II, PL-III, and PL-IV (original data for Figure 6). File S1: Original Western Blot images.
