# Supplementary material for: A Novel Perspective on Lead-Induced Protamine-like Protein-DNA Interactions in Mytilus galloprovincialis: A Molecular and Computational Study
Source: Biomolecules. 2026 Apr 2;16(4):529. doi: 10.3390/biom16040529 (PMC13113032; doi:10.3390/biom16040529)
Supplement: Supplementary file 1 [file biomolecules-16-00529-s001.zip › biomolecules-4183867-Supplementary materials.pdf]

Article

A novel perspective on lead-induced protamine-like protein-DNA interactions in *Mytilus galloprovincialis*: a molecular and computational study

Carmela Marinaro [1,†], Simona Amore [1,†], Rosaria Garofalo [1], Barbara Sebastiano [1], Giulio Santaniello [1], Simona Cafaro [1], Donato Sansone [2], Carmen Di Giovanni [3,*], Gennaro Lettieri [1,4,‡] and Marina Piscopo [1,*,‡]

[1] Department of Biology, University of Naples Federico II, 80126 Naples, Italy
[2] National Reference Centre for the Analysis and Study of Correlations Between the Environment, Animals and Humans, Experimental Zooprophylactic Institute of Southern Italy, Via Salute 2, 80055 Portici, Italy
[3] Drug Discovery Laboratory, Department of Pharmacy, University of Naples Federico II, 80131 Naples, Italy
[4] Department of Life Sciences, Health and Health Professions, Link Campus University, 00165 Rome, Italy

* Correspondence: cdigiova@unina.it (C.D.G.); marina.piscopo@unina.it (M.P.)
† These authors contributed equally to this work.
‡ These authors contributed equally to this work and are co-last.

```
CLUSTAL 2.1 multiple sequence alignment


sp|Q04621|PHI1_MYTED        MPSPTRRSSKSRSKSRSRSRSASASPGKAAKRARSKTPRRGKKRARSPSK
tr|Q1A5Z3|Q1A5Z3_MYTCA      MPSPTRRSSKSRSKSRSRSRSASS-PGKAAKRARSKTPRRGKKRARSPSK
tr|Q9TXE3|Q9TXE3_MYTTR      -PSPTRRSSKSRS--RSRSRSASASPGKAAKRARSKTPRKGKKRARSPSK
                             ************  ********: **************:**********

sp|Q04621|PHI1_MYTED        KARRRSRSTKKTAAKRRKRSSSPKKRRSAGKRRVRAKKKKKK--------
tr|Q1A5Z3|Q1A5Z3_MYTCA      KARRRSRSTKKTAAKRRKRSSSPKKRRSAGKRRVRAEKGGKRRRSRGKKA
tr|Q9TXE3|Q9TXE3_MYTTR      KARRRSRSTKKTAAKRRKRSSSPKKRRSAGKRRVRAKKGGNRRKSRGKKA
                            ************************************:*  ::

sp|Q04621|PHI1_MYTED        ----
tr|Q1A5Z3|Q1A5Z3_MYTCA      AAKK
tr|Q9TXE3|Q9TXE3_MYTTR      AAKK
```

Q04621	Sperm-specific protein Phi-1	Mytilus *edulis*	**92 AA**
Q1A5Z3_MYTCA	Protamine-like PL-III protein	Mytilus *californianus* (California mussel)	**103 AA**
Q9TXE3_MYTTR	Protamine-like protein PL-III	Mytilus *trossulus*	**101 AA**

Figure S1: Multiple sequence alignment of PL-III proteins from *Mytilus* species. The alignment includes the homologous PL-III sequence from *Mytilus californianus* , used as a surrogate for Mytilus galloprovincialis, together with PL-III from *Mytilus trossulus* and the sperm-specific protein Phi-1 from *Mytilus edulis*. Conserved residues are indicated by an asterisk (*), highlighting the high degree of sequence similarity across species, particularly within the putative DNA-binding regions, and supporting the evolutionary conservation of PL proteins within the *Mytilus* genus

Table S1. Key parameters used in the HADDOCK3 molecular docking simulations.

Parameter	Description
**Docking software**	HADDOCK3
**Rigid-body docking**	1000 structures generated with random orientations and energy minimization
**Semi-flexible refinement**	Simulated annealing in torsion-angle space; 200 structures generated
**Final refinement**	Explicit solvent refinement; 200 structures generated
**Force field**	AMBER99SB-ILDN for proteins and DNA
**Water model**	TIP3P water model
**Ligand parametrization**	Pb^{2+} manually parametrized and treated as a non-bonded ligand (Lennard-Jones potential with formal charge)
**Active residues**	Defined based on the basic DNA-binding regions of PL-II and PL-III
**Passive residues**	Neighboring residues surrounding the active sites
**Clustering criteria**	RMSD and fraction of common contacts
**HADDOCK scoring terms**	Electrostatic energy, van der Waals energy, desolvation energy, buried surface area, and AIR contribution
**Cluster selection**	Ten most populated and highest-scoring clusters selected for visual inspection
