# Supplementary figures and images for: A Novel Perspective on Lead-Induced Protamine-like Protein-DNA Interactions in Mytilus galloprovincialis: A Molecular and Computational Study
Source: Biomolecules. 2026 Apr 2;16(4):529. doi: 10.3390/biom16040529 (PMC13113032; doi:10.3390/biom16040529)

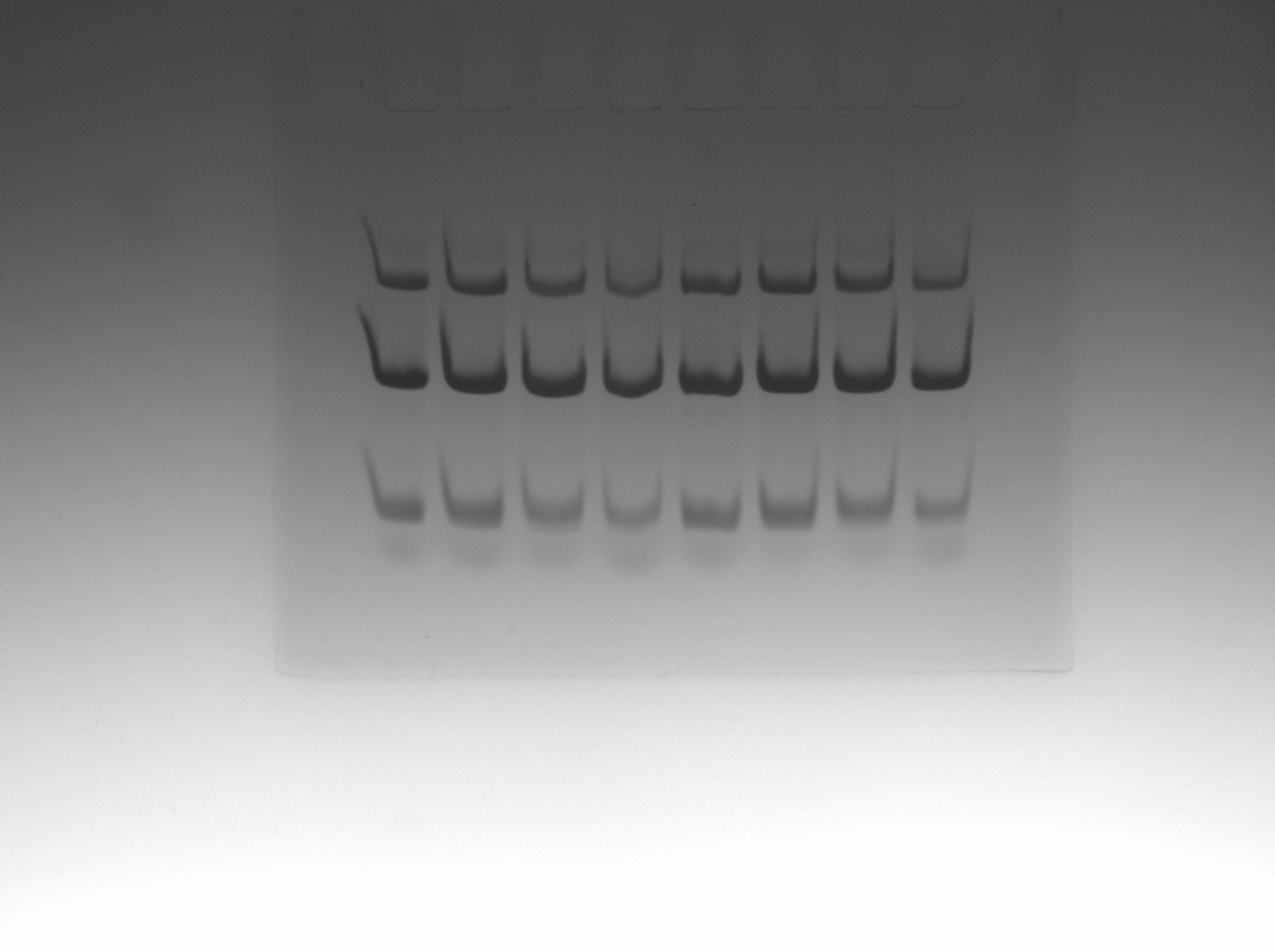

Supplement: Supplementary file 1 [file biomolecules-16-00529-s001.zip › File S1-Original Western Blot image/Figure 1.tif]

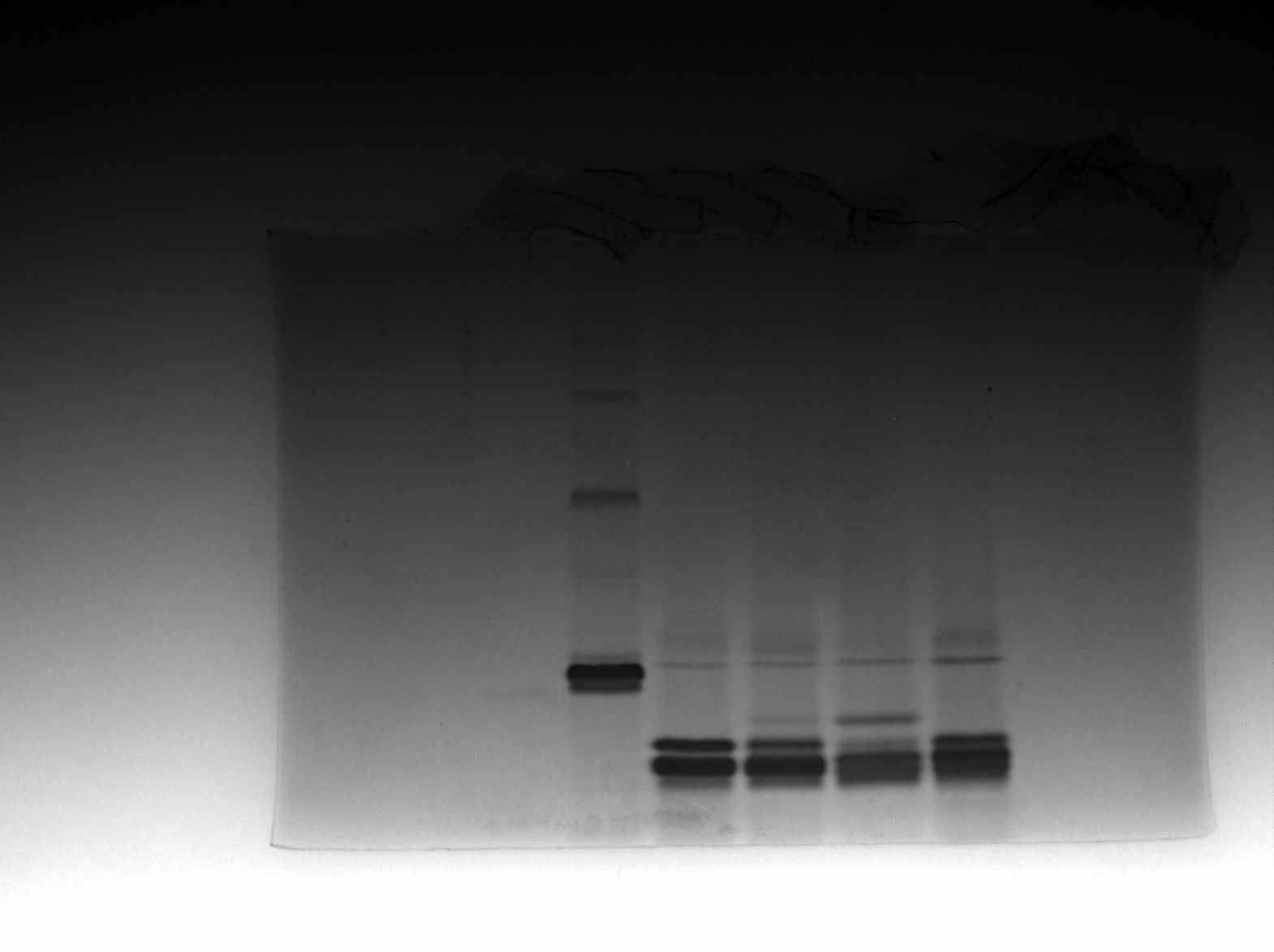

Supplement: Supplementary file 1 [file biomolecules-16-00529-s001.zip › File S1-Original Western Blot image/Figure 2.tif]

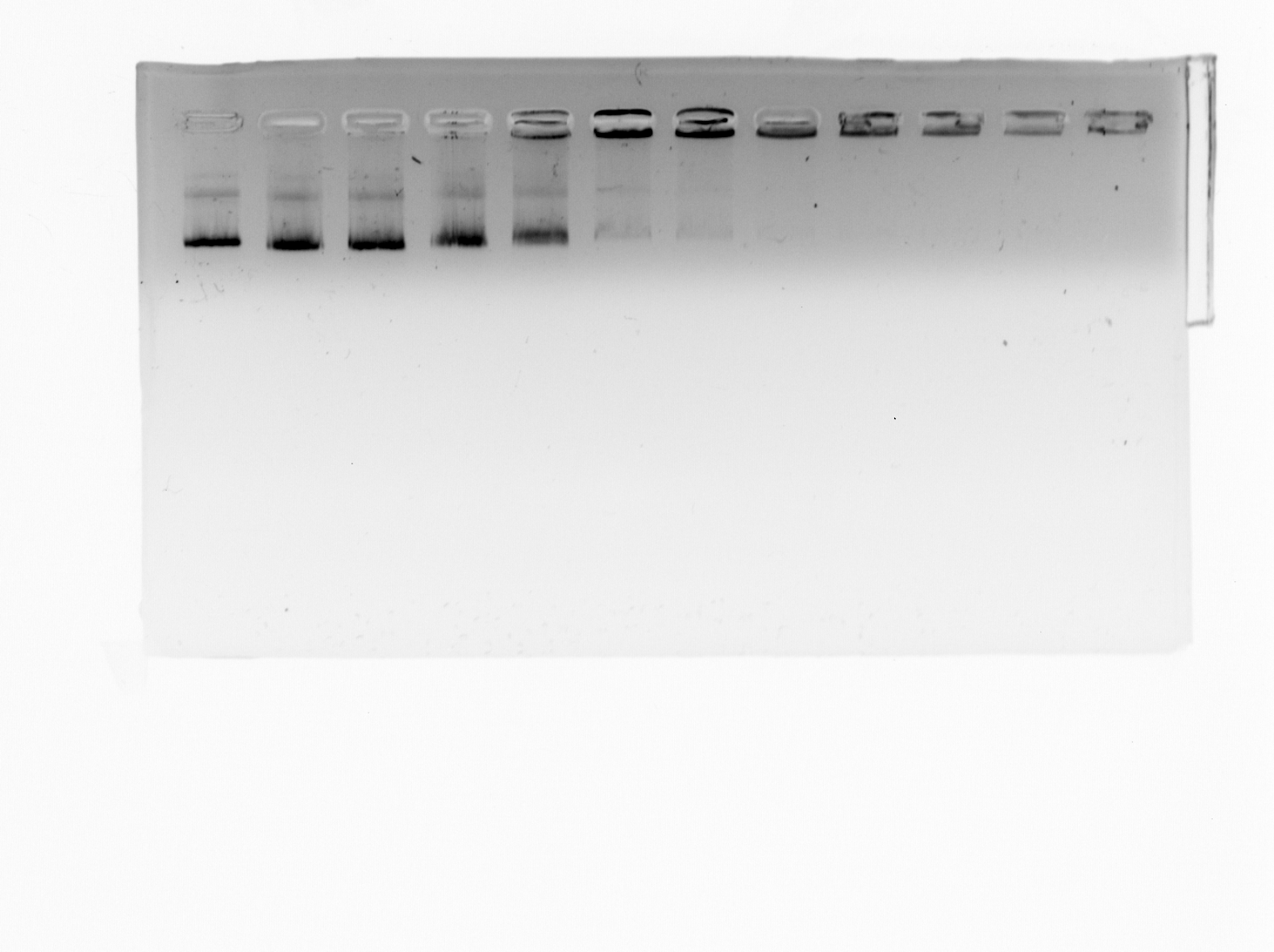

Supplement: Supplementary file 1 [file biomolecules-16-00529-s001.zip › File S1-Original Western Blot image/Figure 3a.tif]

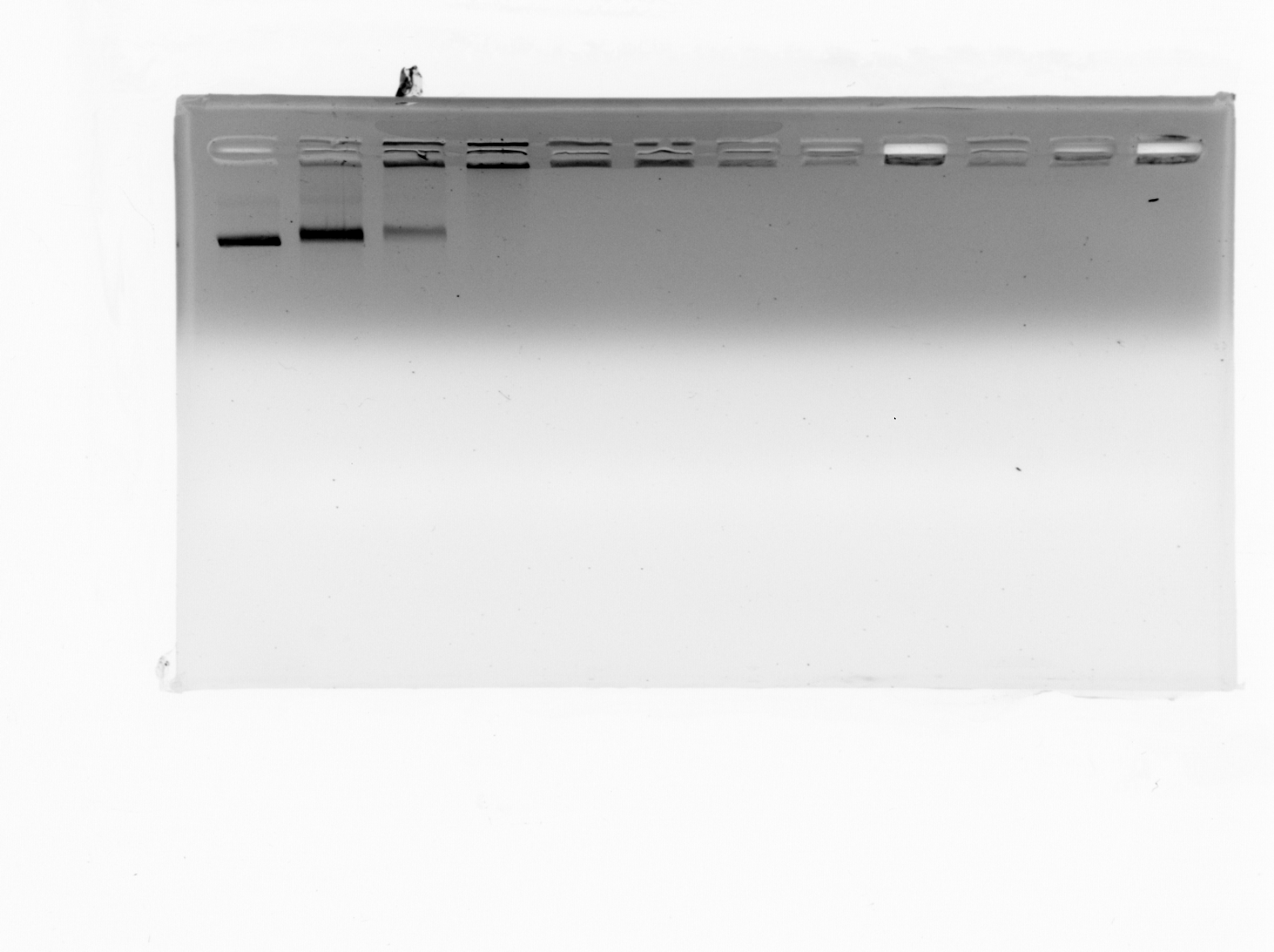

Supplement: Supplementary file 1 [file biomolecules-16-00529-s001.zip › File S1-Original Western Blot image/Figure 3B.tif]

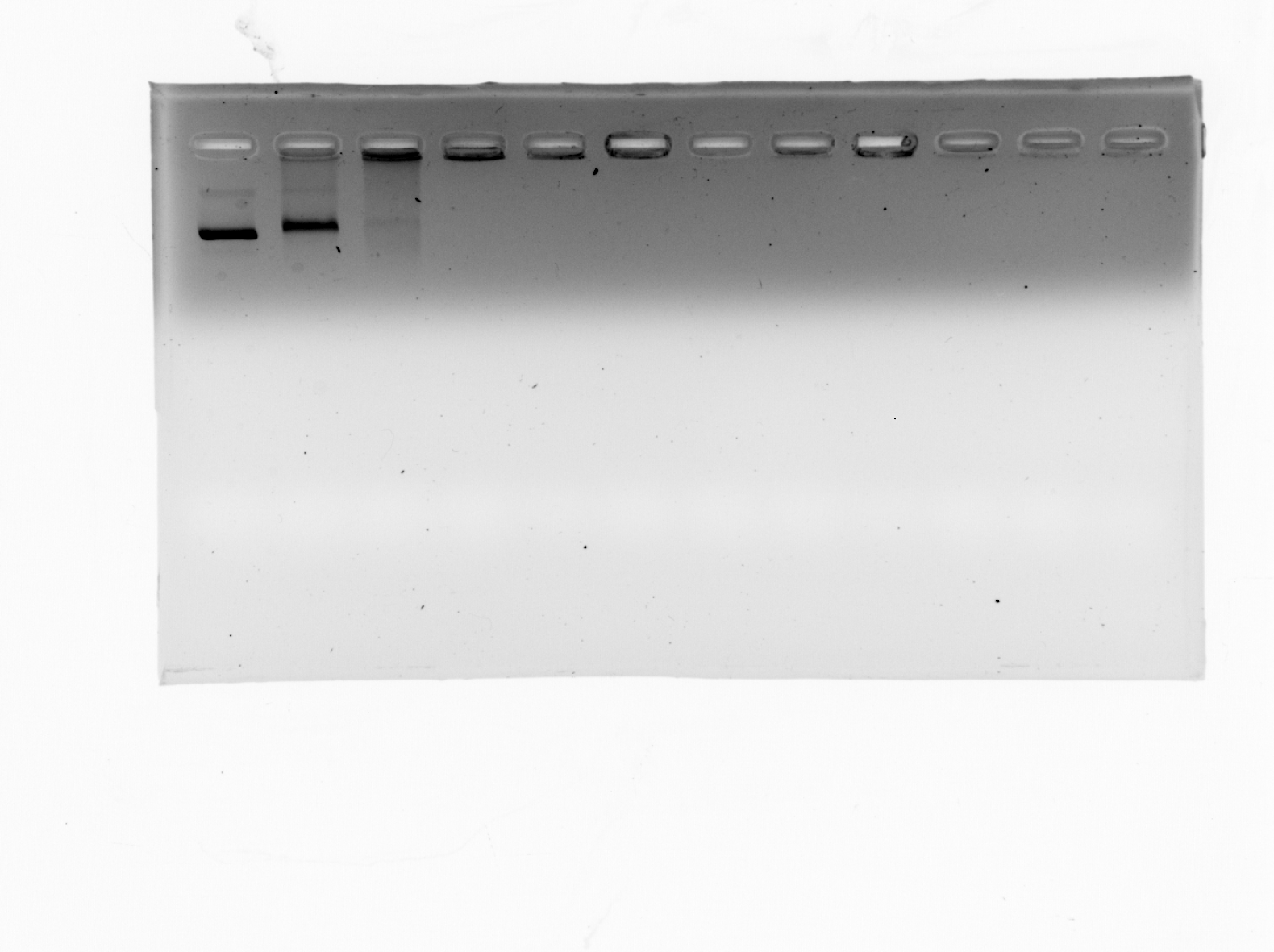

Supplement: Supplementary file 1 [file biomolecules-16-00529-s001.zip › File S1-Original Western Blot image/Figure 3C.tif]

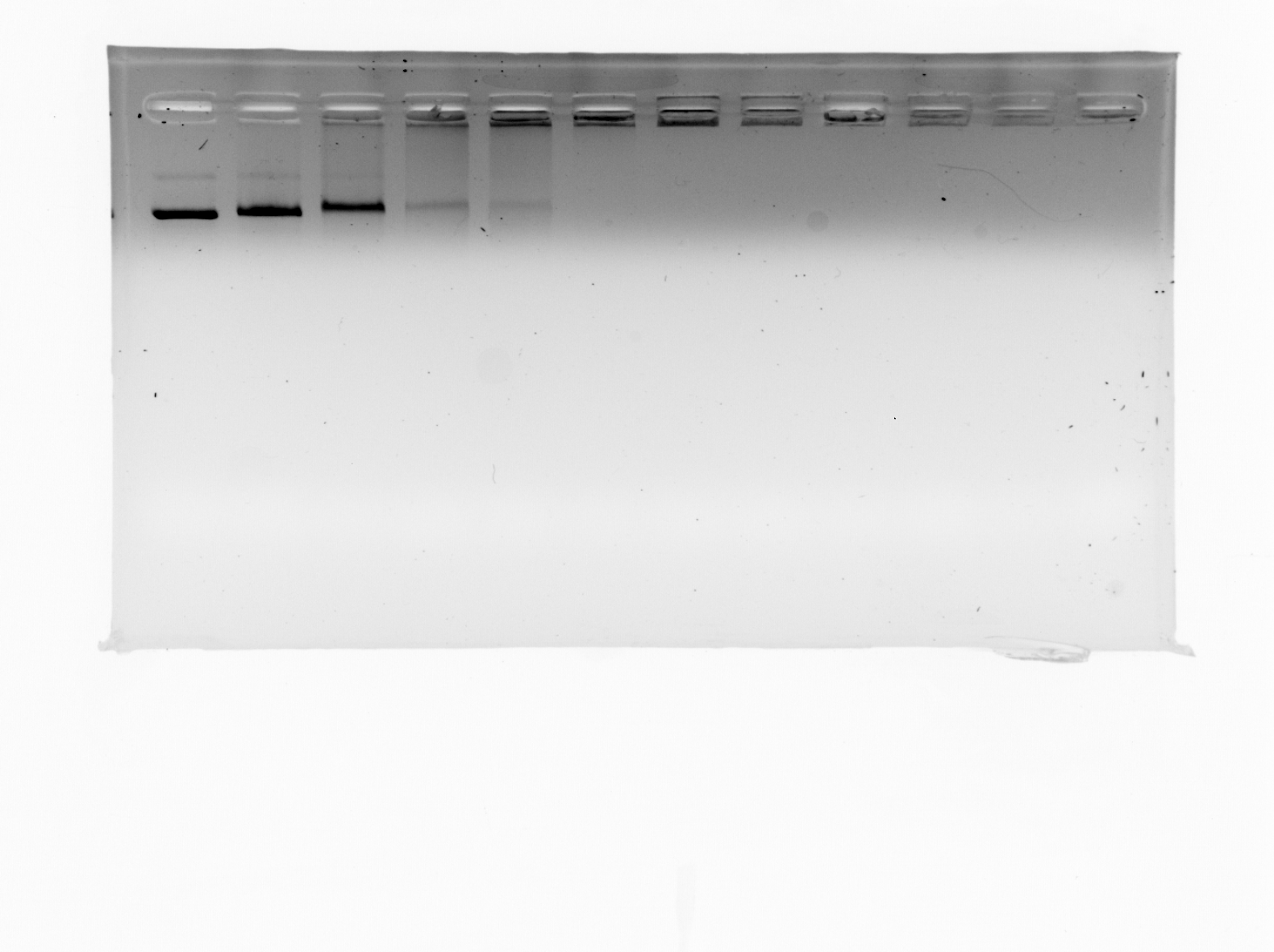

Supplement: Supplementary file 1 [file biomolecules-16-00529-s001.zip › File S1-Original Western Blot image/Figure 3D.tif]

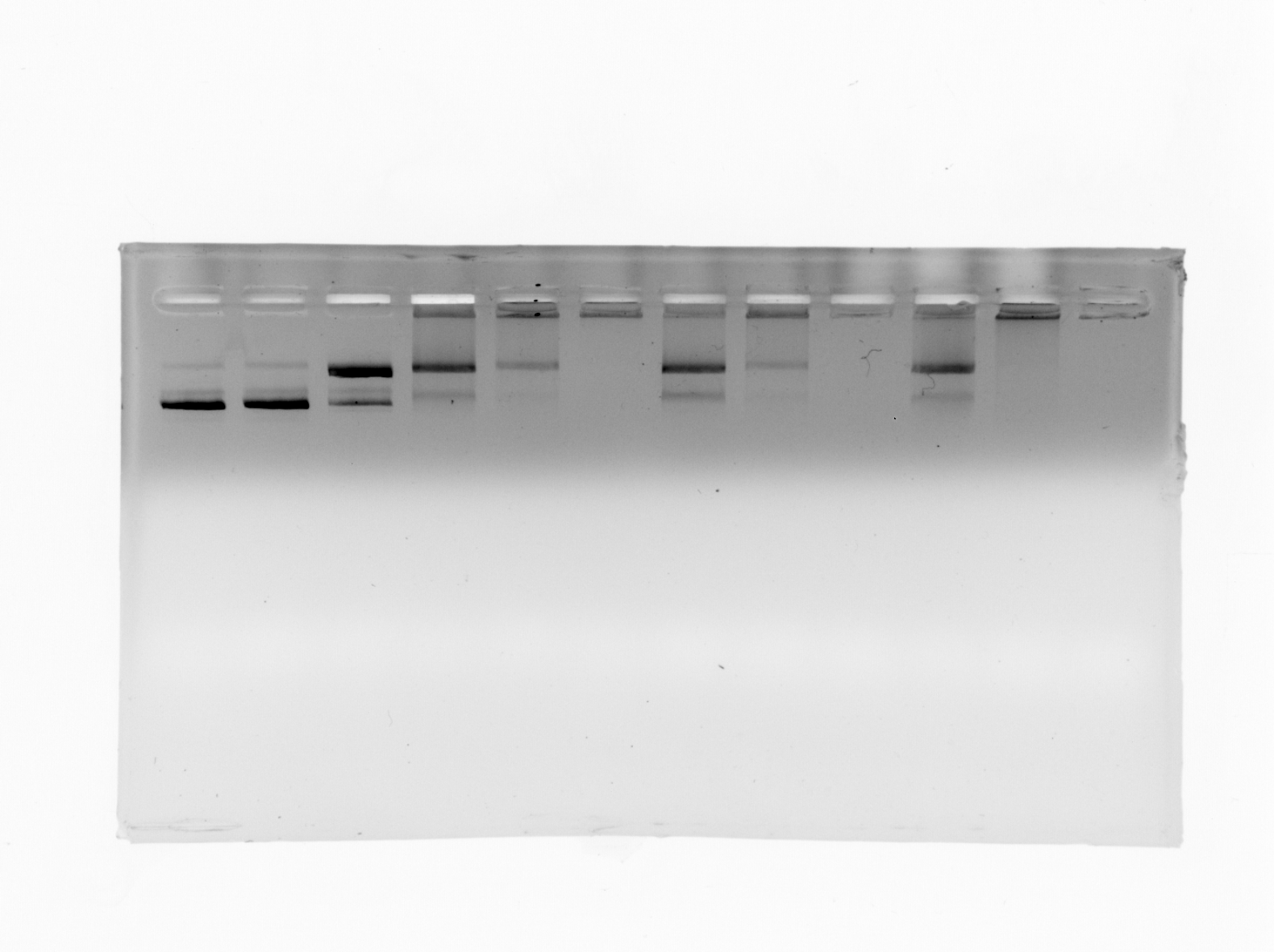

Supplement: Supplementary file 1 [file biomolecules-16-00529-s001.zip › File S1-Original Western Blot image/Figure 4A.tif]

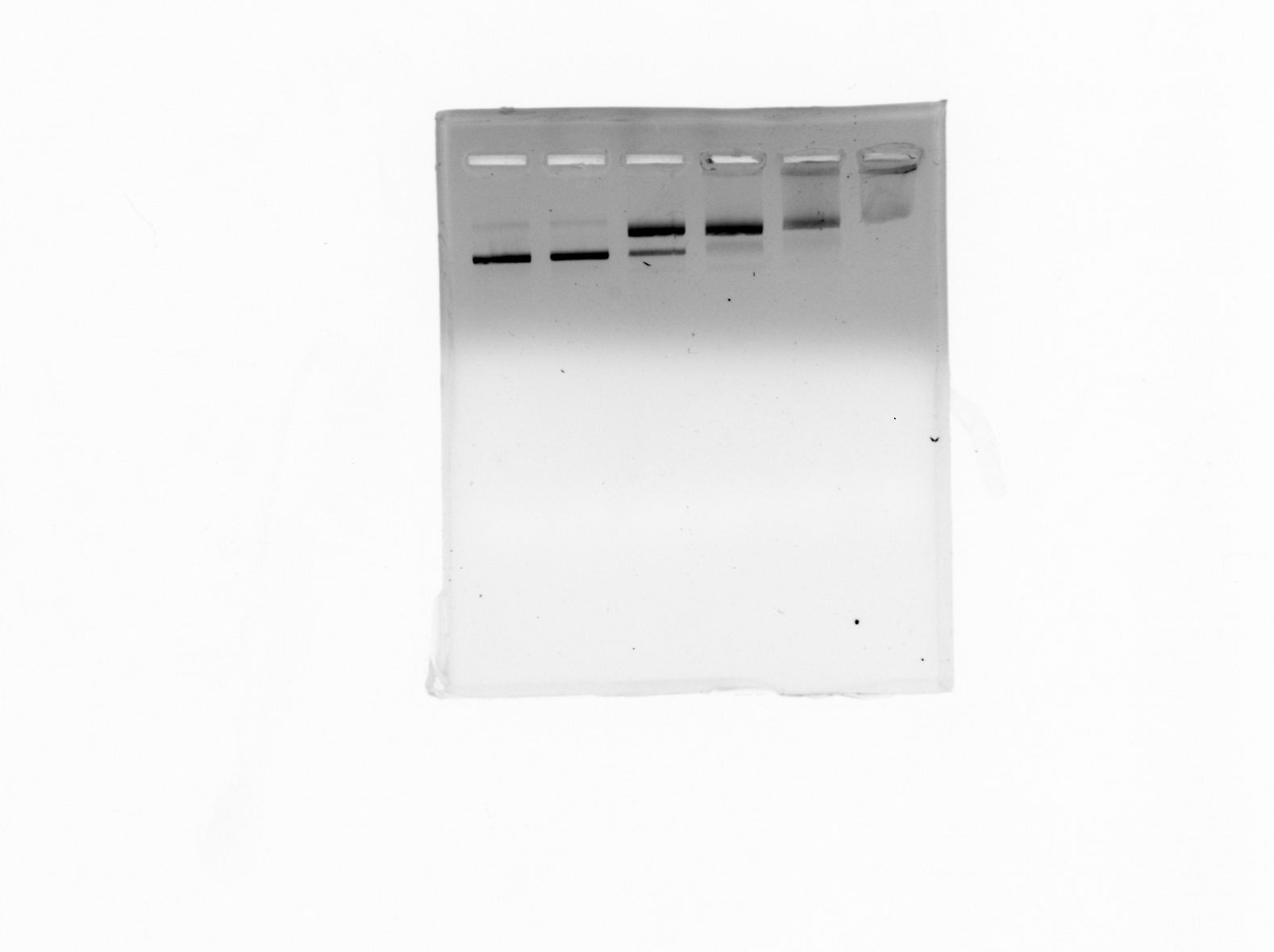

Supplement: Supplementary file 1 [file biomolecules-16-00529-s001.zip › File S1-Original Western Blot image/Figure 4B.tif]
